# Continuous Glucose Monitoring for Women with Gestational Diabetes, CORDELIA: A Multicentric Randomized Controlled Trial, Methodology and Design

**DOI:** 10.3390/jcm15197581

**Published:** 2026-09-29

**Authors:** Ina Geerts, Catherine Yu, Niels Bochanen, Ellen Heyns, Inge Van Pottelbergh, Astrid Morrens, Peter Coremans, Jean-Christophe Philips, Tiphaine Carton, Sara Vandewalle, Joke Marlier, Inge Van Boxelaer, Linsey Winne, Kathleen Bollaerts, Dahae Lee, David Simmons, Arianne Sweeting, Annouschka Laenen, Chantal Mathieu, Katrien Benhalima

**Affiliations:** 1 Department of Chronic Diseases and Metabolism, Clinical and Experimental Endocrinology, KU Leuven, 3000 Leuven, Belgium; ina.geerts@kuleuven.be (I.G.); catherine.yu@student.kuleuven.be (C.Y.); chantal.mathieu@uzleuven.be (C.M.); 2 Department of Endocrinology-Diabetology-Metabolism, Antwerp University Hospital, 2650 Edegem, Belgium; niels.bochanen@uza.be; 3 Department of Endocrinology, General Hospital Groeninge Kortrijk, 8500 Kortrijk, Belgium; ellen.heyns@azgroeninge.be; 4 Department of Endocrinology, AZORG Aalst, 9300 Aalst, Belgium; inge.van.pottelbergh@azorg.be; 5 Department of Endocrinology, ZAS Palfijn, 2170 Merksem, Belgium; astrid.morrens@zna.be; 6 Department of Endocrinology-Diabetology, Vitaz, 9100 Sint-Niklaas, Belgium; peter.coremans@vitaz.be; 7 Department of Diabetology, Nutrition and Metabolic Diseases, CHU de Liège, 4000 Liège, Belgium; jcphilips@chu.ulg.ac.be; 8 Department of Endocrinology, Erasme Hospital, 1070 Brussels, Belgium; tiphaine.carton@hubruxelles.be; 9 Department of Endocrinology-Diabetology, AZ Sint-Jan Brugge, 8000 Brugge, Belgium; sara.vandewalle@azsintjan.be; 10 Department of Endocrinology, UZ Gent, 9000 Gent, Belgium; joke.marlier@uzgent.be; 11 Department of Endocrinology, AZ Sint Lucas Gent, 9000 Gent, Belgium; inge.vanboxelaer@azstlucas.be; 12 Department of Endocrinology, AZ Oostende, 8400 Oostende, Belgium; linsey.winne@azoostende.be; 13 Department of Endocrinology, AZ Sint Maarten Mechelen, 2800 Mechelen, Belgium; kathleen.bollaerts@emmaus.be; 14 Department of Endocrinology, Imelda Bonheiden, 2820 Bonheiden, Belgium; dahae.lee@imelda.be; 15 School of Medicine, Western Sydney University, Sydney, NSW 2560, Australia; da.simmons@westernsydney.edu.au; 16 Department of Endocrinology, Royal Prince Alfred Hospital and Charles Perkins Centre, University of Sydney, Sydney, NSW 2050, Australia; arianne.sweeting@sydney.edu.au; 17 Leuven Biostatistics and Statistical Bioinformatics Centre, KU Leuven, 3000 Leuven, Belgium; annouschka.laenen@kuleuven.be; 18 Department of Endocrinology, University Hospitals Leuven, 3000 Leuven, Belgium

**Keywords:** gestational diabetes mellitus, pregnancy outcomes, continuous glucose monitoring, early gestational diabetes

## Abstract

**Background/Objectives:** Gestational diabetes mellitus (GDM) is associated with increased short- and long-term risks for both mother and child. Screening and treatment between 24 and 30 weeks’ gestation reduces adverse pregnancy outcomes, while emerging evidence suggests that earlier diagnosis and treatment (<20 weeks’) in high-risk populations may further improve neonatal outcomes. Currently, self-monitoring of blood glucose (SMBG) remains the standard of care for management but provides only intermittent glycemic data and limited insight into overall glucose patterns. Real-time continuous glucose monitoring (rt-CGM) has transformed management in pregnancies complicated by type 1 diabetes but has shown conflicting results in GDM; it is thus unclear whether similar benefits may extend to women with GDM, including women with early GDM. **Methods:** The Continuous Glucose Monitoring for Women With Gestational Diabetes (CORDELIA) study is an ongoing, large, open-label, multicenter randomized controlled trial conducted across 14 centers in Belgium and two centers in Australia. Women diagnosed with GDM are randomized in a 1:1 ratio to either rt-CGM (intervention group) or standard care with SMBG (control group). Both women with early GDM diagnosed before 20 weeks’ gestation and those diagnosed between 24.0 and 29.6 weeks’ gestation are included. Participants attend the following visits: baseline visit at GDM diagnosis, an additional visit at 20.0–23.9 weeks’ (if early GDM), 31.0–33.6 weeks’ and 36.0–38.6 weeks’, and a postpartum oral glucose tolerance test (OGTT) assessment (6.0–23.6 weeks after delivery). At each visit, clinical assessment, HbA1c, and (blinded) CGM are evaluated, alongside validated patient-reported outcome questionnaires. The primary objective is to compare the incidence of a composite of adverse pregnancy outcomes between the rt-CGM and SMBG groups. The primary outcome is a composite of adverse pregnancy outcomes including preterm delivery <37 weeks, LGA [customized percentiles according to Gestation Related Optimal Weight chart (GROW)], neonatal hypoglycemia requiring intravenous dextrose, neonatal intensive care unit (NICU) admission > 24 h, neonatal respiratory distress, stillbirth or neonatal death, phototherapy, birth trauma, and shoulder dystocia. Secondary outcomes include maternal glycemia, insulin use, pregnancy and neonatal outcomes, glycemic variability, patient-reported outcomes, and prespecified secondary analyses according to timing of GDM diagnosis and maternal BMI. **Trial Registration:** ClinicalTrials.gov: NCT06310356. First posted: 15 March 2024.

## 1. Introduction

Gestational diabetes mellitus (GDM) is defined as diabetes first diagnosed during pregnancy, below the threshold of overt diabetes [1]. GDM increases the risk of several short- and long-term complications including large gestational age (LGA) infants [2]. LGA infants face higher risks of shoulder dystocia and cesarean delivery, and GDM is further associated with higher risks of preeclampsia, neonatal hypoglycemia, and neonatal respiratory distress [3]. Furthermore, women with GDM have a higher risk of developing future type 2 diabetes, along with increased risk of metabolic syndrome and cardiovascular disease [4,5]. Offsprings of women with GDM face elevated risks of obesity and type 2 diabetes in the long term [6]. A systematic review and meta-analysis of nearly 4000 women demonstrated that screening and treatment of GDM between 24 and 28 weeks of gestation reduces several adverse outcomes, including primary cesarean delivery, shoulder dystocia, macrosomia, LGA, NICU admission and reduced risk of birth injury [7]. Additionally, the Treatment Of Booking Gestational diabetes Mellitus (TOBOGM) trial showed that screening and treatment of early GDM (<20 weeks) in 802 women with risk factors reduced the risk of adverse pregnancy outcomes, largely driven by fewer cases of neonatal respiratory distress compared to deferred treatment [8]. Initial management of maternal glycemia includes medical nutrition therapy, physical activity and self-monitoring of blood glucose (SMBG) based on American Diabetes Association (ADA) criteria [fasting glucose < 95 mg/dL (<5.3 mmol/L), 1 h postprandial glucose < 140 mg/dL (<7.8 mmol/L), and/or 2 h <120 mg/dL (<6.7 mmol/L)] [1]. If glycemic targets are not achieved within 1–2 weeks, pharmacotherapy, preferably insulin, is generally recommended [9].

While SMBG remains the standard of care, real-time continuous glucose monitoring (rt-CGM) offers a more detailed view of glycemic patterns. The Continuous glucose monitoring in pregnant women with type 1 diabetes (CONCEPTT) trial showed that rt-CGM use in pregnant women with type 1 diabetes resulted in improved HbA1c, increased time in range (TIRp), and reduced glycemic variability, which translated into improved neonatal outcomes, compared with SMBG [10]. However, evidence supporting rt-CGM use in women with GDM remains conflicting and with very limited data in women with early GDM. Recently, larger randomized controlled trials (RCTs) have examined rt-CGM use in GDM with inconsistent findings. The Continuous glucose monitoring in the management of gestational diabetes in Switzerland (DipGluMo) trial (n = 302) found no reduction in its composite adverse pregnancy outcome and even reported lower TIRp in the rt-CGM group at 34–38 weeks’ gestation [11]. The Glycemic control and pregnancy outcomes with real-time continuous glucose monitoring in gestational diabetes (GRACE) trial (n = 375) reported a significantly lower incidence of their primary outcome, LGA, in the intervention group (4% vs. 10%). However, overall LGA rates were lower than generally expected, while small-for-gestational-age (SGA) rates were numerically higher in the CGM group, with high insulin use in both groups, suggesting potential risk of overtreatment [12]. A smaller RCT in the US (NCT04605497), not powered on pregnancy outcomes, showed improved TIRp with rt-CGM (93% vs. 88%), but concurrent SMBG was needed for safety given the study was undertaken prior to Food and Drug Administration (FDA) clearance of CGM use in pregnancy [13]. Taken together, the results across the studies remain conflicting, highlighting the need to improve our knowledge on the impact of CGM for the management of GDM. Furthermore, all these RCTs only included women with late GDM with no evidence regarding the use of CGM in early GDM, a group at higher risk of adverse outcomes [14]. The CORDELIA trial will therefore provide important insights into the effectiveness of rt-CGM in women with GDM and assess whether its benefits differ across clinically relevant subgroups, including early versus late GDM.

### Objectives

The introduction of CGM has transformed the management of pregnancies complicated by type 1 diabetes. However, it remains unclear whether these benefits extend to women with GDM. The CORDELIA trial was therefore designed to evaluate whether rt-CGM, compared with SMBG, improves a composite of adverse pregnancy outcomes, glycemic control and other maternal and neonatal outcomes among women with both early and late GDM.

**The primary objective** is to compare a composite of adverse pregnancy outcomes between the rt-CGM group and the SMBG group, including preterm delivery < 37 weeks, LGA (customized percentiles according to GROW), neonatal hypoglycemia requiring intravenous dextrose, neonatal intensive care unit (NICU) admission > 24 h, neonatal respiratory distress, stillbirth or neonatal death, phototherapy, birth trauma, and shoulder dystocia.

The choice of the components of the composite outcome was primarily based on the TOBOGM trial [8], which, at the time of study design, was the only RCT with a large cohort of women diagnosed with early GDM. In addition to the outcomes reported in TOBOGM, NICU admission and neonatal hypoglycemia were included because these outcomes are clinically relevant and are important drivers of the healthcare costs associated with pregnancy complications.

**Secondary objectives are exploratory:**

**Main secondary outcomes:**

- **Hierarchical win ratio** analysis of the composite of adverse pregnancy outcomes ranking the components of the composite as follows: Stillbirth and neonatal death, shoulder dystocia, birth trauma, preterm birth, NICU admission, respiratory distress, neonatal hypoglycemia, LGA, and phototherapy;
- **Time in range** (day, night) between 63 and 140 mg/dL (3.5–7.8 mmol/L) and 63 and 120 mg/dL (3.5–6.7 mmol/L);
- **Time above range** (day, night) for thresholds above 140 mg/dL (>7.8 mmol/L) and above 120 mg/dL (>6.7 mmol/L);
- **Gestational weight gain (kg);**
- **SGA infants according to customized birth weight standards (GROW, gestation.net).**

**Exploratory subgroup analyses:** Differential effectiveness of rt-CGM between clinically relevant subgroups, including early versus late GDM.

**Exploratory glycemic targets:** Time spent between (day, night) [70 and 95 mg/dL (3.9–5.3 mmol/L) and 54 and 95 mg/dL (3.0–5.3 mmol/L)]. Time in hypoglycemia [<50 mg/dL (<2.8 mmol/L), <54 mg/dL (<3.0 mmol/L), <63 mg/dL (<3.5 mmol/L), and <70 mg/dL (<3.9 mmol/L)]. Mean glucose, area under the curve (AUC), glycemic variability, HbA1c and glucose management indicator (GMI) during pregnancy and postpartum. To evaluate the feasibility and clinical relevance of tighter glycemic targets, including TIR < 120 mg/dL (<6.7 mmol/L) and mean glucose < 100 mg/dL (<5.6 mmol/L), based on evidence from pregnancies with normal glucose tolerance [15].

**Exploratory lifestyle and behavior:** To evaluate the impact of rt-CGM on lifestyle and behavioral factors by assessing dietary intake, physical activity, sleep duration, and sleep patterns during pregnancy.

**Exploratory maternal and neonatal outcomes:** To evaluate gestational hypertension, preeclampsia, and the incidence of SGA infants according to local reference curves.

**Exploratory patient-reported outcomes:** To evaluate treatment satisfaction, quality of life, and fear of hypo- and hyperglycemia.

**Healthcare utilization and cost-effectiveness:** To evaluate the cost-effectiveness of rt-CGM compared with SMBG.

**Postpartum outcomes:** Attention will be given to differences in the risk of postpartum glucose intolerance between women with early and late GDM. In addition, CGM-derived metrics will be explored as potential predictors of postpartum glucose intolerance, with the aim of determining whether CGM could serve as an alternative to the postpartum oral glucose tolerance test (OGTT) for identifying women at increased risk of persistent dysglycemia after pregnancy.

The health economic evaluation and the postpartum analyses are planned as separate scientific publications. Therefore, detailed methodologies, outcome definitions, and statistical analysis plans for these prespecified secondary objectives will be described and reported separately.

## 2. Materials and Methods

### 2.1. Study Design and Setting

**Figure 1:**
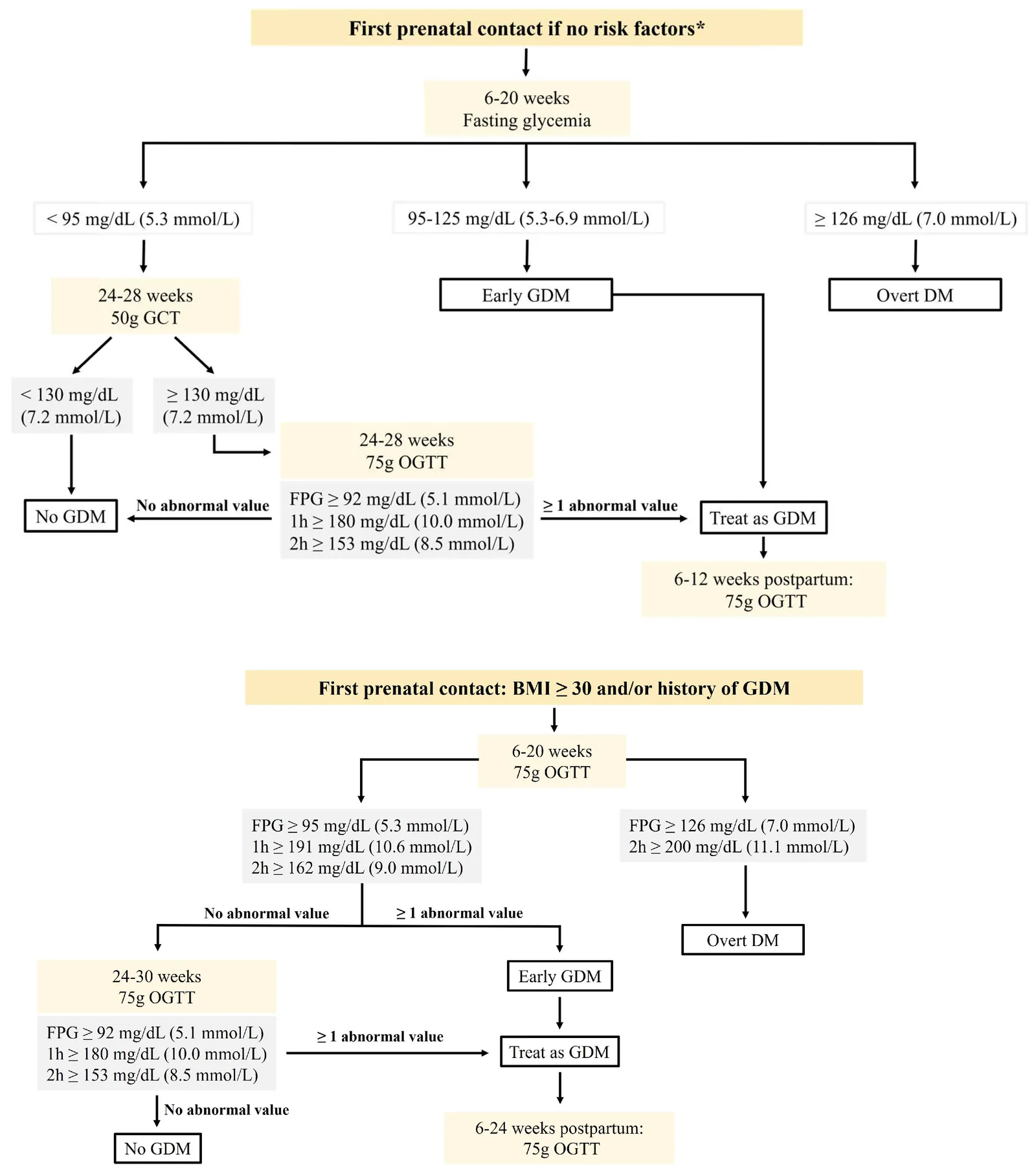
The 2024 Flemish screening consensus for GDM. GDM: gestational diabetes mellitus, GCT: glucose challenge test, OGTT: oral glucose tolerance test, FPG: fasting plasma glucose, DM: diabetes mellitus. * BMI ≥ 30 and/or history of GDM, adapted from Benhalima et al. [16].

The CORDELIA trial is a Belgian Australian multicenter, open-label RCT conducted in 16 centers. The study evaluates the effect of rt-CGM compared with standard SMBG in women with GDM, diagnosed either early (<20 weeks’ gestation) or later in pregnancy (24.0–29.6 weeks’ gestation) following the 2024 Flemish screening consensus (Figure 1) [16].

Following screening and informed consent, participants undergo collection of clinical, demographic and medical history data, first-trimester ultrasound information, and glucose monitoring data. Clinical assessments include measurement of weight and blood pressure, routine HbA1c assessment, and completion of self-administered questionnaires. All participants complete a 7-day run-in phase before randomization while wearing a blinded CGM (Freestyle Libre^®^ Pro IQ (Abbott Diabetes Care Ltd., Witney, UK)) and performing SMBG four times daily (OneTouch Ultra Plus Reflect^®^ (LifeScan, Oevel, Belgium) in Belgium, and in line with routine practice, Accu-Chek^®^ Performa (Roche Diabetes Care Australia Pty Limited, Sydney, Australia) in Australia). Participants are randomized 1:1, stratified by timing of GDM diagnosis and BMI, to rt-CGM (Freestyle Libre^®^ 3+ (Abbott Laboratories, Kilkenny, Ireland)) or standard SMBG care.

### 2.2. rt-CGM Intervention Group

Participants randomized to rt-CGM receive standardized education from experienced diabetes educators and the study team before starting the device. Education covers device use, sensor placement and replacement, and interpretation of glucose values and trends with a TIRp of 63–140 mg/dL (3.5–7.8 mmol/L) aiming for ≥90% TIRp. Participants are instructed to record the start of each meal in the CGM application, either using the meal marker or a free-text note, to facilitate interpretation of postprandial glucose responses and trends in relation to meal timing. Insulin initiation and titration are based on ADA fasting and 1 or 2 h postprandial glucose values, the overall clinical profile, and, as an additional measure of glycemic control, CGM-derived TIRp [1]. Furthermore, alarms for signal loss and hyperglycemia are recommended. All participants using rt-CGM are also provided with a backup glucometer and are instructed to use capillary SMBG in case of suspected inaccurate CGM readings, device malfunction, or other technical problems. A new sensor is placed every 14 days. They are encouraged to use the device at least 80% of the time and continue CGM until the first few days postpartum after delivery (until discharge from the hospital). CGM data from the day of delivery and the early postpartum period will be analyzed separately. Glucose values are monitored using a smartphone app (iOS or Android), or, if the participant does not have a compatible smartphone, a reader is provided.

### 2.3. Standard SMBG Control Group

Women in the control group receive standard care with SMBG following ADA criteria [1], with fingerstick measurements 4 times daily (fasting and 1 h or 2 h after each meal), and wear a blinded CGM for 14 days following every study visit to match with the 14-day window of the intervention group. The blinded CGM is started in the hospital and if a sensor is removed before the end of the prespecified period, participants are asked to contact the study team; as the protocol specifies a minimum of 80% CGM data completeness, a new sensor can be placed. Possible incomplete data will be handled as described in the statistical analysis plan. The blinded CGM data are not used to inform clinical management or treatment decisions. SMBG results are reviewed at clinical assessments and are used for treatment decisions according to the predefined glycemic targets and insulin treatment algorithm.

Both groups receive identical standard GDM management according to ADA-based clinical practice, including lifestyle counseling (dietary guidance, physical activity, and gestational weight targets) and uniform glycemic targets [fasting < 95 (<5.3 mmol/L), 1 h < 140 (<7.8 mmol/L), and/or 2 h < 120 mg/dL (<6.7 mmol/L)] [1]. Insulin therapy will be initiated for persistent hyperglycemia despite lifestyle measures, after excluding transient factors such as stress, poor sleep, special occasions, or infection. Basal insulin, preferably glargine U100, will be initiated in the evening if ≥2 fasting glucose values are ≥95 mg/dL (5.3 mmol/L) within one week. Rapid-acting insulin (aspart, lispro, or ultra-rapid analogues) will be initiated if ≥2 postprandial values are ≥140 mg/dL (7.8 mmol/L) at 1 h or ≥120 mg/dL (6.7 mmol/L) at 2 h within one week. Glargine will be started at 3–10 IU and increased by 2–4 IU; for doses >25 IU, a 20% increase may be used. Rapid-acting insulin will be started at 2–8 IU and increased by 2–4 IU if targets are not achieved within 3–14 days. Alternative basal insulins include glargine U300, degludec, or intermediate-acting neutral protamine Hagedorn (NPH) insulin. Women requiring insulin will have standardized follow-up every two weeks by the endocrinologist. Metformin is not used in Belgian centers (due to potential concerns about the long-term metabolic risks for the offspring), while it may be used selectively in Australian sites [16].

**Figure 2:**
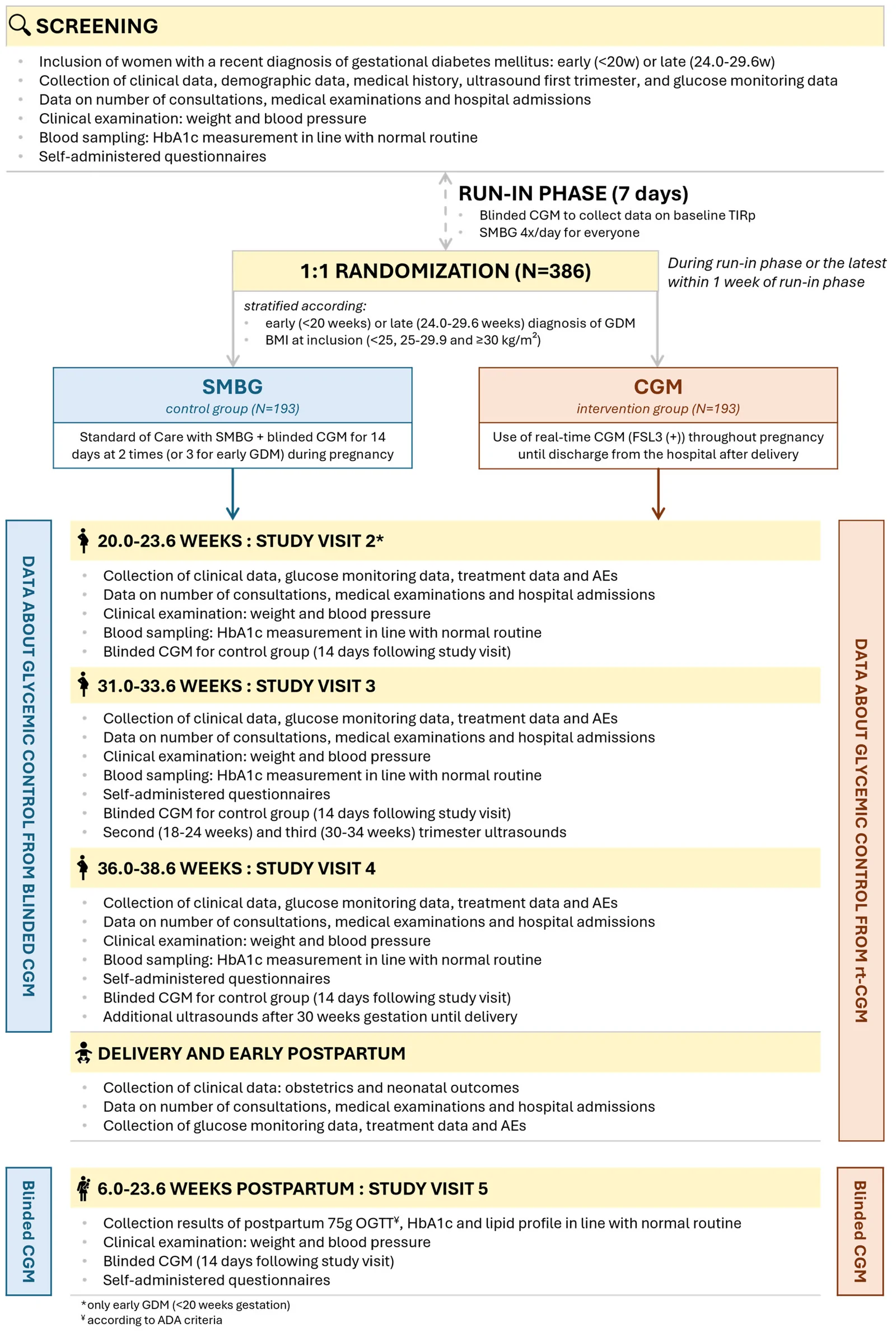
Study design overview. CGM: continuous glucose monitoring, OGTT: oral glucose tolerance test, SMBG: self-monitoring of blood glucose, AE: adverse event.

Follow-up visits are scheduled at study visit (SV) 2: 20.0–23.6 (only for early GDM), SV3: 31.0–33.6, and SV4: 36.0–38.6 weeks’ gestation. At each visit, clinical data, glucose monitoring data, treatment information, adverse events, healthcare utilization, weight, blood pressure, and routine HbA1c measurements are collected. Self-administered questionnaires are completed at SV3 and SV4, and ultrasound data are collected according to routine clinical practice. In Australia, study visits are performed remotely via digital consultations. No additional study visit is scheduled for delivery or during the early postpartum period. Pregnancy, delivery and neonatal outcomes, treatment data, CGM data of the intervention group and adverse events are collected from participants’ electronic medical records by researchers on site who are aware of group allocation. No blind endpoint adjudication committee will be used. A final postpartum visit (SV5) takes place between 6.0 and 23.6 weeks after delivery and includes assessment of a 75 g 2 h OGTT, HbA1c and lipid profile according to routine care, clinical examination, placement of the blinded CGM for 14 days, and completion of questionnaires. The study design is illustrated in Figure 2.

### 2.4. Recruitment and Eligibility

Recruitment in Belgium took place at five university and nine large non-university hospitals: UZ Leuven, UZ Gent, UZA, CHU Liège, Erasme, Azorg-Aalst, AZ Groeninge Kortrijk, AZ St Maarten Mechelen, AZ St Lucas Gent, ZAS Palfijn, Imelda Bonheiden, VITAZ St Niklaas, AZ St Jan Brugge, and AZ Oostende. In Australia, recruitment was undertaken at Royal Prince Alfred Hospital and Campbelltown Hospital (Sydney).

Women meeting the criteria of overt diabetes [fasting plasma glucose ≥ 126 mg/dL (7.0 mmol/L), HbA1c ≥ 6.5%, and/or 2 h OGTT ≥ 200 mg/dL (11.1 mmol/L)] are excluded [16].

GDM screening follows the 2024 Flemish screening consensus [16]. Early screening (<20 weeks) is based on fasting plasma glucose for women without any risk factors, as early OGTT is considered burdensome and reserved for high-risk women (BMI ≥ 30 kg/m^2^ and/or history of GDM). Diagnosis of early GDM is based on higher cut-offs compared to late GDM (based on the TOBOGM trial) [8,16]. Women without early GDM are screened at 24–30 weeks using either a two-step approach (glucose challenge test followed by OGTT if abnormal) or a one-step 75 g OGTT. GDM is diagnosed according to the International Association of Diabetes and Pregnancy Study Groups (IADPSG) criteria: fasting 92–125 mg/dL (5.1–6.9 mmol/L), 1 h ≥ 180 mg/dL (10.0 mmol/L), or 2 h ≥ 153 mg/dL (8.5 mmol/L) (Figure 1) [17].

Potential participants were recruited during patient consultations and/or education sessions at one of the participating sites in the endocrinology department, using flyers in Dutch, French, or English. In addition, posters were available in the waiting rooms of the different sites. Women received study information within two weeks of GDM diagnosis, and, if interested, provided written informed consent. Table 1 summarizes the eligibility criteria for the CORDELIA trial.

### 2.5. Randomization

Participants in this multicenter study are allocated to either treatment group using central block randomization (block size 4) in a 1:1 allocation ratio, stratified by early versus late GDM (<20 weeks or 24–30 weeks of gestation) and baseline BMI (<25, 25–29.9, or ≥30 kg/m^2^). The randomization sequence was generated by the study statistician using computer-generated random numbers (SAS PROC PLAN) and uploaded to the eCRF, REDCap [19,20].

### 2.6. Modifications to the Study Protocol

Two protocol amendments were implemented to improve feasibility and recruitment without compromising study integrity.

Amendment 1: Approved by the Ethics Committee (EC) Leuven on 24 September 2024, it added four study sites prior to study initiation: Imelda Hospital Bonheiden, CHU Erasme, CHU de Liège, and UZ Gent. Study documentation was updated according to the requirements of the ethics committees of these centers.

Amendment 2: The second amendment, approved by the EC Leuven on 24 April 2025, added two international study sites: Campbelltown Hospital (South Western Sydney Local Health District) and Royal Prince Alfred Hospital (Sydney Local Health District) in Sydney, Australia. These centers were selected for their expertise in early GDM, as demonstrated in the TOBOGM trial [8]. Study procedures were discussed with the principal investigators of the Australian centers to ensure adherence to the study protocol and standardization across countries. Follow-up is conducted according to the same protocol at all sites, ensuring consistent data collection, including for the cost-effectiveness analysis. Potential differences between countries and centers will be accounted for in both the statistical and cost-effectiveness analyses. Study documentation was updated accordingly.

The initial study protocol (Version 2), approved on 6 August 2024, was subsequently amended through Protocol Version 3 (24 September 2024) and Protocol Version 4 (24 April 2025). As detailed above, these amendments were solely related to the addition of participating study centers and the corresponding administrative updates to study documentation. No changes were made to the study objectives, eligibility criteria, interventions, outcomes, sample size assumptions, or statistical analysis methods.

The statistical analysis plan was prespecified in the initial study submission and approval on 6 August 2024 and remained unchanged throughout the study. Recruitment was completed at 386 participants in June 2026 with 362 participants in Belgium and 16 in Australia. After randomization, 194 participants were included in the tr-CGM group, and 191 participants were included in the SMBG group. One participant ended the study before randomization. A blinded sample size review was conducted on 16 February 2026 after approximately 50% of the primary outcome data had become available and did not result in any modifications to the protocol, statistical analysis plan, or planned analyses.

### 2.7. Data Collection

A detailed overview of all data collection procedures is provided in Table 2.

Primary outcome: A composite of adverse pregnancy outcomes: preterm delivery < 37 weeks, LGA (according to ethnic group and sex adjusted customized percentiles for birth weight GROW [gestation.net]), neonatal hypoglycemia (requiring intravenous dextrose), NICU admission (>24 h), neonatal respiratory distress (at least 4 h of respiratory support with supplemental oxygen, CPAP, or intermittent positive-pressure ventilation during the first 24 h after delivery), stillbirth or neonatal death, phototherapy for jaundice, birth trauma and shoulder dystocia. Hence, the primary outcome is a binary variable, taking the value 1 if at least one of the adverse pregnancy outcomes occurs, or zero otherwise.

Secondary outcomes assessed during pregnancy, delivery, and in the early postpartum period:

Glycemic outcomes: Mean blood glucose (over 24 h period, during the day and night 00–06 separately), glucose AUC, TIR between 70 and 95 mg/dL (3.9–5.3 mmol/L) during the day, TIR overnight (00:00–06:00) between 70 and 95 mg/dL (3.9–5.3 mmol/L), TIR 63–140 mg/dL (3.5–7.8 mmol/L) overnight (00:00–06:00), TIR 63–140 mg/dL (3.5–7.8 mmol/L) during the day, TIR 54–95 mg/dL (3.0–5.3 mmol/L) during the day and night, TIR 63–120 mg/dL (3.5–6.7 mmol/L) overnight (00:00–06:00), TIR 63–120 mg/dL (3.5–6.7 mmol/L) during the day and night, TIR 70–120 mg/dL (3.9–6.7 mmol/L) and TIR 70–140 mg/dL (3.9–7.8 mmol/L) the during day and overnight, and number of participants who met the TAR > 140 mg/dL (>7.8 mmol/L) < 5% criterium. Percentage of time < 54 mg/dL (<3.0 mmol/L) in line with ADA recommendations for hypoglycemia definition (outside pregnancy), as well as time < 50 mg/dL (<2.8 mmol/L), <63 mg/dL (<3.5 mmol/L), and <70 mg/dL (<3.9 mmol/L). Standard deviation (SD), coefficient of variation (CV), time overnight > 100 mg/dL (>5.6 mmol/L), mean glycemia with target 98 mg/dL (5.4 mmol/L), postprandial hyperglycemia [time > 120 mg/dL (>6.7 mmol/L) at 2 h and time > 140 mg/dL (>7.8 mmol/L) at 1 h], and time > 180 mg/dL (>10.0 mmol/L), HbA1c, and glucose management indicator (GMI) during pregnancy and postpartum.

Maternal outcomes: Time to start insulin, insulin need and dose, (excessive) gestational weight gain according to National Academy of Medicine (NAM) criteria, postpartum hemorrhage, cesarean sections (planned and emergency), instrumental delivery, perineal injury, labor inductions, preeclampsia, gestational hypertension, and miscarriage (<20 weeks’).

Neonatal outcomes: Macrosomia (>4 Kg and >4.5 Kg), SGA (according to ethnic group and sex adjusted customized percentiles for birth weight GROW [gestation.net]), SGA [according to local Flemish customized growth curves SPE rapport 2009], birth weight (percentile), low birth weight (<2500 g), head circumference and crown–heel length, birth weight > percentile 97, fetal malformation, polycythemia, neonatal hypoglycemia < 40 mg/dL (<2.2 mmol/L), and Apgar scores (0–5 and 10 min).

The individual components of primary composite outcomes: Preterm delivery < 37 weeks, LGA (according to ethnic group and sex adjusted customized percentiles for birth weight [gestation.net]), LGA [SPE rapport 2009], neonatal hypoglycemia, NICU admission and duration, respiratory distress, stillbirth or neonatal death, phototherapy for jaundice, birth trauma and shoulder dystocia, duration of maternal hospitalization stay postpartum, treatment satisfaction, quality of life, fear of hypo- and hyperglycemia, dietary intake, physical activity, sleep duration, and experience with CGM use.

Health economic outcomes: Use of healthcare system [number of appointments (face-to-face or virtual) and duration with physician, diabetes nurses, dieticians…] and the cost-effectiveness of the intervention. No multiplicity adjustment is applied and all analyses beyond the primary objectives are exploratory.

Standardized definitions used for maternal pregnancy outcomes according to IADPSG [21]: Duration of pregnancy, insulin regimen and dose, gestational weight gain, length of hospital stay, type of labor (spontaneous, inducted or caesarean before labor); type of delivery (vaginal route, forceps or vacuum, caesarean section during labor or planned caesarean section); postpartum hemorrhage (requiring or offered blood transfusion +/− an operative procedure); gestational hypertension (≥20 weeks of gestation: BP ≥ 140/90 mmHg); chronic hypertension (hypertension diagnosed prior to pregnancy or during pregnancy before 20 weeks’ gestation); preeclampsia [≥20 weeks of gestation: new onset of hypertension and proteinuria or the new onset of hypertension and significant end-organ dysfunction with or without proteinuria (dipstick ≥ 2+, ≥0.3 g protein/24 h or ≥30 mg/dL protein in spot urine or spot urine protein/creatinine ratio ≥ 30 mg protein/mmol creatinine)]; HELLP syndrome (Hemolysis with a microangiopathic blood smear, Elevated Liver enzymes, and a Low Platelet count (<100,000/microliter); perineal injury, perineal injury (3rd or 4th degree perineal laceration); shoulder dystocia (vaginal cephalic delivery that requires additional obstetric maneuvers to deliver the fetus after the head has delivered and gentle traction has failed); birth trauma: spinal cord injury, peripheral nerve injury/brachial plexus, basal skull fracture or depressed skull fracture, clavicular fracture; long bone fracture (humerus, radius, ulna, femur, tibia or fibula); and cranial hemorrhage (subdural or intracerebral of any kind [confirmed by cranial ultrasound, computerized tomography scan, or magnetic resonance imaging]).

Ultrasound data collection: Obstetric ultrasound data collection performed during pregnancy according to the International Society of Ultrasound in Obstetrics and Gynecology (ISUOG) practice guidelines [22,23,24]: date of the ultrasound with gestational age; estimated due date; indication(s) (e.g., routine first-trimester exam, second-trimester exam, or third-trimester exam); presence of (normal) fetal heart activity with heart rate [in beats per minute (bpm)] and (normal) fetal movements; fetal position; placental position; fetal biometry [crown–rump length (CRL in mm), biparietal diameter (BPD), occipitofrontal diameter (OFD), head circumference (HC), transcerebellar diameter (TCD), abdominal circumference (AC and percentiles), femur length (FL), BPD/OFD, HC/AC, and BPD/FL]; estimated fetal weight (EFW) or fetal size with indication of the used formula; calculation of EFW according to the Hadlock [25,26] formula using AC, HC, and FL with BPD; EFW percentile (customized or uniform); fetal LGA (EFW or AC > percentile 90) and fetal SGA (EFW or AC < percentile 10); (early or late) fetal growth restriction (FGR: with deliberate narrow definition of EFW < percentile3); intracranial translucency; presence of (normal) nasal bone; nuchal translucency (NT) thickness (in mm); assessment of fetal anatomy and detection of fetal structural anomalies according to gestational age [e.g., visualization and assessment of head, cranium and brain, face and neck, thorax, heart (4-chamber view, outflow tract views, 3-vessel view, 3-vessel trachea view, situs), abdomen and/or abdominal wall, spine, limbs, extremities, stomach and intestines, bladder, kidneys, urinary tract and genitalia]; the amount of amniotic fluid (normal vs. abnormal) with measurement of the amniotic fluid index (AFI) (oligohydramnios is defined as AFI < 5 cm and polyhydramnios as AFI > 25 cm) and evaluation of amniotic membranes; assessment of (3-vessel) umbilical cord and cord insertion into placenta; screening for preeclampsia (determined by the calculator of the Fetal Medicine Foundation to predict the risk of preeclampsia at 11.0 to 13.6 weeks); cervix length measurement (in mm) with evaluation of presence of funneling; non-invasive prenatal testing (NIPT) results if consent provided to perform this test; and fetoplacental Doppler with evaluation of pulsatility index (PI) of the uterine artery and/or umbilical artery (left, right and/or mean).

Secondary outcomes at the postpartum visit: Duration of breastfeeding, type of contraception, lipid profile, the glycemic metrics for non-pregnant populations [such as TIR 70–180 mg/dL, time below range and time above range, glycemic variability measures (SD, CV), CGM predictors for glucose intolerance based on the OGTT and values of prediabetes and diabetes according to the ADA] will be evaluated based on the blinded CGM [1].

Clinical measurements: Consistent with routine clinical assessment. Height is measured on the first visit. On every visit to the diabetes outpatient clinic, blood pressure (BP) and weight are measured. BMI is calculated as Kg/m^2^.

Lab measurements: Follow routine local practice and are analyzed at each center. HbA1c is measured at the OGTT for GDM diagnosis and subsequently every 4–6 weeks and at the postpartum OGTT. All participating laboratories are accredited according to national quality standards.

Self-administered questionnaires: A self-designed questionnaire on general habits (including smoking and alcohol habits) and socio-economic factors (such as education, income and ethnicity), as used in the BEDIP-N study [27]. Food frequency questionnaire (FFQ) validated for the Belgian and suitable for the Australian population [28]; the international questionnaire on physical activity (IPAQ) validated for use in the Belgian and Australian population [27,29]; quality of life (SF-36) [30] and the EQ-5D-5L questionnaire [31]; the 20-item Center for Epidemiologic Studies-Depression (CES-D) questionnaire [32]; validated six-item short-form STAI questionnaire on anxiety [33]; the Hypoglycemia Fear Survey II (HFS-II) [34]; the Diabetes Treatment Satisfaction Questionnaire (status), which measures satisfaction with diabetes treatment regimens in people with diabetes [35]; and the Diabetes Treatment Satisfaction Questionnaire (change), which has been developed to overcome potential ceiling effects (i.e., where respondents score maximum or near-maximum satisfaction at baseline and can show little or no improvement at follow-up) [36]. For women with GDM using rt-CGM: the Glucose Monitoring Experiences Questionnaire (GME-Q) [37], the validated Pittsburg sleep quality index to evaluate sleep quality [38] and self-designed questionnaire on fear of hyperglycemia, a self-designed questionnaire on breastfeeding and contraception postpartum as used in the BEDIP-N study [27], and a self-designed questionnaire to evaluate the preference of test postpartum (comparing CGM with OGTT).

Downloading of glucose measurements: The CGM data used for routine GDM monitoring are downloaded via the Abbott LibreView platform or provided as CSV files/PDFs in the database. Study personnel at each participating center have access only to the CGM data of their own participants, while the central study team at UZ Leuven has access to all glycemic data from all participants for central glycemic analyses. These data are retrieved and used centrally at UZ Leuven for data analysis. In addition, the UZ Leuven study team centrally processes data from the blinded CGM. Therefore, each local study team provides the blinded glucose sensors to UZ Leuven. Several calculations of the measured glucose values (such as different ranges of time in target, time below range and time above range) by the CGM might also be processed centrally by the sensor company (Abbott) and then provided to the coordinating study team in Leuven. SMBG data from glucometers are digitally uploaded via the LifeScan OneTouch Reveal^®^ platform by the Belgian study teams. For the Australian centers, SMBG data are digitally uploaded for analysis by the sponsor. Abbott and LifeScan have no access to identifiable personal data. All data are used exclusively for this study and not for any other (commercial) purposes.

### 2.8. Discontinuation of Participation

In the event of early discontinuation from the study, whether due to withdrawal of consent, a significant medical reason, initiation of medication with a substantial impact on glucose metabolism, a change in the participant’s condition that justifies discontinuation of treatment, or noncompliance (e.g., failure to attend study visits), data collection through the electronic patient form and medical records may continue if the participant provides additional informed consent for ongoing data collection. Otherwise, no further data will be collected after discontinuation.

## 3. Statistics

All analyses will follow the intention-to-treat principle, meaning that patients will be analyzed according to their original treatment assignment. The primary and all secondary analyses will be performed at a two-sided 5% significance level.

### 3.1. Sample Size

Based on the pregnancy outcomes in the BEDIP-N study [39] (which is based on a Belgian population with GDM), and data for stillbirth and phototherapy based on the TOBOGM trial [8], we estimated a total prevalence of the composite of adverse pregnancy outcomes of 60% (preterm delivery: 7.4%, LGA: 12.2%, neonatal hypoglycemia: 14%, NICU admissions: 14%, shoulder dystocia: 0.9%, birth trauma: 0.4%, neonatal respiratory distress: 0.9%; stillbirth or neonatal death: 0.5%, and phototherapy: 11%). The primary outcome is a binary composite of neonatal outcomes, taking value 1 if at least one of the adverse neonatal events is present, or 0 otherwise.

The sample size was determined for a two-sided chi-square test to demonstrate a difference in the primary outcome between the two treatment groups, assuming a difference of 15 percent points (45% in the CGM vs. 60% in the control group). At a 5% two-sided significance level, with 80% power and estimated dropout of 10%, the sample size was estimated at 386. The estimated difference in primary outcome is based on previous studies and estimations (NCT03981328 and NCT05037526) [40,41,42]. When the study was designed, the results of the recently published large RCTs evaluating the use of CGM in women with GDM were not yet available [11,12]. Moreover, this is the first trial to include a large prespecified population with early GDM, representing 34% of the total study population. Therefore, at the time of study design, it was considered plausible that the rate of adverse pregnancy and neonatal outcomes in our study population could be higher than that subsequently observed in these CGM trials, given the substantial proportion of participants with early GDM. Furthermore, a blinded sample size review was performed after approximately 50% of participants had primary outcome data available, with an observed pooled composite rate of 31.3%. Assuming an equal absolute effect size of 15% points difference between the treatment arms, this would indicate a smaller sample size than initially calculated. At the observed event rate, this corresponds to a relative risk reduction of approximately 39%. Consequently, smaller treatment effects would not be detected with adequate power. Furthermore, at present, 310 deliveries are reported in our eCRF. Data are unavailable for 4.6% of deliveries due to loss to follow-up, early termination without signing the second ICF, or pregnancy loss. This proportion is well below the 10% dropout rate anticipated in the original sample size calculation, and we therefore expect to remain within the predefined 10% dropout allowance. As the protocol allowed only for an increase, and not a reduction, no further sample size adjustments were made.

### 3.2. Data Analysis

A logistic regression model will be used for the primary endpoint, with the binary composite as the response variable and treatment arm as the main explanatory variable. Both stratification factors (early vs. late GDM and BMI category) and country will be included as covariates. Center will be included as a random effect, with 16 of the 386 participants recruited in Australian centers. Results will be reported by an odds ratio with a 95% confidence interval. Following the intention-to-treat principle, all patients will be analyzed according to the treatment arm in which they were randomized. To account for dropout, missing outcome data will be handled using multiple imputation. A fully conditional specification approach will be used to generate 10 complete datasets. The imputation model will include treatment arm, both stratification factors (early vs. late GDM and BMI category), center, country, and the following auxiliary variables: baseline HbA1c, BMI, and gestational age at diagnosis. The analysis will be performed on each complete dataset, and the 10 results will be combined following multiple imputation principles [43]. In addition, sensitivity analyses will be conducted to assess the robustness of conclusions under departures from the missing-at-random assumption.

Continuously and cross-sectionally measured secondary outcomes will be analyzed using linear mixed models with treatment arm as the main explanatory variable. Both stratification factors (early vs. late GDM and BMI category) and country will be included as covariates. Center will be included as a random effect. Results will be presented as means and mean differences with 95% confidence intervals. Longitudinally measured continuous outcomes will be analyzed using linear mixed models with participant as an additional random effect. Time measurement will be included in the fixed effects model, in addition to treatment arm, stratification factors and country. Transformation will be considered for continuous outcomes if necessary to meet distributional assumptions. Binary and cross-sectionally measured secondary outcome variables will be analyzed using logistic regression models. Results will be presented as odds ratios with 95% confidence intervals. Longitudinally measured binary outcome variables will be analyzed using logistic regression with an additional random effect for participant and including time of measurement in the analysis model. Time to initiate insulin therapy will be analyzed using a Cox proportional hazards regression model, with time from randomization to start of insulin therapy as the outcome and treatment arm as the main explanatory variable. For this analysis, participants will be censored at the date of their last study assessment if they withdraw from the study, are lost to follow-up, or do not initiate insulin therapy before delivery by the end of the follow-up period. Both stratification factors (early vs. late GDM and BMI category) and country will be included as covariates. Center will be included as a random effect (frailty term). Results will be reported as hazard ratios with 95% confidence intervals. Kaplan–Meier curves will be presented for each treatment arm. All secondary analyses will be performed according to the intention-to-treat principle in the sense that participants will be analyzed in the treatment arm according to randomization, irrespective of treatment compliance. Missing data will be dealt with by available case analysis if the amount of missing data is modest (≤5%). If the missing data rate is >5%, multiple imputations will be applied using the same methods as described for the primary endpoint.

In addition to the primary composite outcome analysis, a hierarchical win ratio analysis will be performed as a secondary analysis, with the components ranked according to clinical importance, giving priority to more severe outcomes (stillbirth and neonatal death, shoulder dystocia, birth trauma, preterm birth, NICU admission, respiratory distress, neonatal hypoglycemia, LGA, and phototherapy). Furthermore, each individual outcome will be reported separately in the final trial manuscript.

As prespecified sub-analyses, the primary and secondary outcomes will be compared between the group with early GDM and the group with GDM diagnosed later in pregnancy. A descriptive analysis will compare the primary and secondary outcomes between the group treated with insulin and the group treated with lifestyle measures alone. As insulin initiation occurs after randomization and may be influenced by treatment allocation, these analyses are susceptible to selection bias and will be interpreted with caution. In addition, as prespecified sub-analyses, we will also compare the primary and secondary outcomes across different age categories (<25, 25–29, 30–34, 35–40, >40 years) and BMI categories (<25, 25–29.9 and ≥30 Kg/m2). Subgroup analyses will be conducted using tests for interaction between treatment arm and subgroup. These analyses are exploratory, as the study is not powered to detect subgroup-specific effects.

Statistical analyses will be performed by statistician A. Laenen (PhD statistics KU Leuven) using SAS software (version 9.4 of the SAS System for Windows). A. Laenen has extensive experience in studies related to GDM. As an exploratory analysis, a per-protocol analysis will be performed based on participants with ≥80% of the time use of the FSL3 (+) for the intervention arm and with ≥80% of the time use of SMBG for the control arm. The trial statistician remains blinded until database lock.

### 3.3. Patient and Public Involvement: Patients Were Not Directly Involved in the Design, Conduct, Reporting, or Dissemination Plans of This Study

**Trial management:** This is an academic research trial. University Hospitals Gasthuisberg Leuven (UZ Leuven) is the sponsor and has responsibility for the overall management of the study.

**Data management:** Each site opened following an initial initiation visit. Throughout the study, the coordinating investigator conducts periodic quality assurance visits to ensure protocol adherence. The first visit to each site occurred within six months of the first participant inclusion, followed by annual visits and a close-out visit at study completion. The coordinating investigator may review pseudonymized source documents to verify the accuracy of data recorded in the CRFs. Investigators and relevant personnel must be available for quality assurance visits, audits, or inspections, dedicating sufficient time to these processes. To ensure confidentiality, each participant is assigned a unique subject identification number, and all data are referenced using these identifiers only. All data collected are entered into and managed in REDCap [19,20]. Trained staff at each participating site first record the coded data on standardized paper worksheets and subsequently enter them into REDCap in accordance with applicable local guidelines [19,20]. Glucose monitoring and insulin therapy data are collected via the manufacturer’s cloud software. Blinded CGM data are not used for treatment decisions, and treating physicians do not have access to the primary outcome data of the trial. Although the study is open-label, this approach helps minimize the potential for treatment-related bias in the primary outcome.

**Ethics and dissemination:** The study was approved by the Ethics Committees of UZ Leuven with Belgian registration number B3222024001490 (s68870) and by the Australian EC, South Western Sydney Local Health District Human, with reference number 2025/ETH00656, and is conducted in accordance with the Declaration of Helsinki. Recruitment started in mid-November 2024 and was completed in June 2026. A completed SPIRIT 2025 checklist can be found in the Supplementary Materials.

## Figures and Tables

**Table 1:** Eligibility criteria of the CORDELIA trial.

Inclusion Criteria	Exclusion Criteria
Voluntary written informed consent of the participant has been obtained before any screening procedures	The patient has a new diagnosis of overt diabetes or a history of type 2 or type 1 diabetes, or the presence of auto-immune antibodies for T1DM (such as GAD, ICA and IA-2)
At least 18 years of age at the time of signing the Informed Consent Form (ICF)	A physical or psychological disease likely to interfere with the conduct of the study (based on the assessment by the treating physician based on the medical record and/or clinical examination)
Singleton pregnancy	Use of medications at study inclusion with a significant impact on glycemia (such as high-dose glucocorticoids and diabetes medication such as metformin)
GDM diagnosed by the fasting glycemia (95–125 mg/dL) or 75 g OGTT (FPG 95–125 mg/dL; 1 h ≥ 191 mg/dL, 2 h 162–199 mg/dL) between 6.0 and 19.6 weeks or diagnosed by the IADPSG/2013 WHO criteria [17,18] (fasting ≥ 92 mg/d 1 h ≥ 180 mg/d or 2 h ≥ 153 mg/d based on 75 g OGTT between 24.0 and 29.6 weeks of pregnancy)	Participation in an interventional trial with an investigational medicinal product (IMP) or device
Needs to be able to understand and speak Dutch, French or English	Multiple pregnancy
Have email access	History of bariatric surgery
	Known allergy to the adhesives used with the CGM

GDM: gestational diabetes mellitus, OGTT: oral glucose tolerance test, WHO: World Health Organization, IADPSG: International Association of Diabetes and Pregnancy Study Groups, CGM: continuous glucose monitoring.

**Table 2:** Detailed study overview.

Procedures/Assessment	Screening	Run-in Phase	Randomization					
Visits/Contacts	Visit 1	Contact	Contact	Visit 2 *	Visit 3	Visit 4	Delivery	Visit 5
Timing (weeks)	For early GDM: 6.0–19.6 weeks For late GDM: 24.0–29.6 weeks	Start at screening or at the latest within 2 weeks of screening	Start procedure during run-in phase or at the latest within 1 week of run-in week	20.0–23.6 weeks	31.0–33.6 weeks	36.0–38.6 weeks	Delivery	6–23.6 weeks postpartum
Informed consent	X ^1^							
Eligibility criteria	X							
Demographics	X							
Medical history	X							
Physical examination, in line with normal routine	X			X	X	X		X
Standardized education on lifestyle and SMBG	X							
Randomization			X					
Education on use of CGM for intervention group			X					
HbA1c (analyzed locally in line with normal routine)	X			X	X	X		X
75 g OGTT and lipid profile (or fasting glycemia with HbA1c if OGTT not feasible), analyzed locally in line with normal routine								X
**Use of study device**				X	X	X		
Blinded CGM for 1 week		X						
Blinded CGM for 2 weeks **				X	X	X		X
Downloading glucose monitoring data and treatment data				X	X	X		
Data collection pregnancy outcomes and early postpartum							X	
Q on general habits and socio-economic factors	X							
Frequency food Q	X					X		X
International Q on physical activity	X					X		X
SF-36 Q	X					X		X
EQ-5D-5L Q	X					X		X
The 20-item Center for Epidemiologic Studies-Depression (CES-D) Q	X					X		X
Validated six-item short-form STAI Q on anxiety	X					X		X
Hypoglycemia Fear Survey II					X	X		
Q on fear of hyperglycemia					X	X		
The diabetes treatment satisfaction Q (status)					X	X		
The diabetes treatment satisfaction Q (change)						X		
The validated Pittsburg sleep quality index	X					X		X
*** Glucose, monitoring experiences Q						X		
Q to evaluate the user-friendliness of the CGM compared to OGTT postpartum								X
Q on breastfeeding and anticonception								X
Compliance assessment				X	X	X		
(Serious) adverse event assessment	X	X	X	X	X	X	X	X
Concomitant medication	X		X	X	X	X	X	X

I: Informed consent process takes place prior to all other study-related procedures at the screening visit. * Visit 2 is only applicable for women with early GDM (<20 weeks). ** Blinded CGM after randomization is only for control group during pregnancy, but for all participants at the postpartum visit. *** Only for the intervention group with CGM. X ^1^ provides important extra information about the ICF. Q: questionnaire, CGM; continuous glucose monitoring, OGTT: oral glucose tolerance test, SMBG: self-monitoring of blood glucose.

## Data Availability

Requests for access to study data will be considered where appropriate. Inquiries for data should be addressed to the principal investigator, Prof. Katrien Benhalima.

## Supplementary Materials

The following supporting information can be downloaded at: https://www.mdpi.com/article/10.3390/jcm15197581/s1, ICF on data collection by dropout after randomization, general ICF, and the SPIRIT 2025 check-list. Reference [44] is cited in Supplementary Materials.

## Abbreviations

The following abbreviations are used in this manuscript:

GDM	Gestational diabetes mellitus
LGA	Large-for-gestational age
TIR	Time in range
CGM	Continuous glucose monitoring
RCT	Randomized controlled trial
HbA1c	Glycated hemoglobin
TBR	Time below range
TAR	Time above range
SD	Standard deviation
CV	Coefficient of variation
BEDIP-N	Belgian Diabetes in Pregnancy Study
CONCEPTT	Continuous glucose monitoring in pregnant women with type 1 diabetes
TOBOGM	Treatment Of Booking Gestational diabetes Mellitus
GRACE	Glycemic control and pregnancy outcomes with real-time continuous glucose monitoring in gestational diabetes
DipGluMo	The Continuous glucose monitoring in the management of gestational diabetes in Switzerland
HFS-II	Hypoglycemia Fear Survey II
SF-36	36-Item Short-Form Health Survey
CES-D	20-item Centre for Epidemiologic Studies-Depression
FFQ	Food frequency questionnaire
DTSQ	Diabetes Treatment Satisfaction Questionnaire
HELLP	Hemolysis, Elevated Liver enzymes and Low Platelets
SGA	Small-for-gestational age
CPAP	Continuous positive airway pressure
OGTT	Oral glucose tolerance test
BMI	Body mass index
NICU	Neonatal intensive care unit
FDA	Food and Drug Administration
ADA	American Diabetes Association
SMBG	Self-monitoring of blood glucose
IADPSG	International Association of Diabetes and Pregnancy Study Groups
GROW	Gestation Related Optimal Weight
AUC	Area under the curve
GMI	Glucose management indicator
NAM	National Academy of Medicine
ISUOG	International Society of Ultrasound in Obstetrics and Gynecology
CRL	Crown–rump length
BPD	Biparietal diameter
OFD	Occipitofrontal diameter
HC	Head circumference
TCD	Transcerebellar diameter
AC	Abdominal circumference
FL	Femur length
EFW	Estimated fetal weight
FGR	Fetal growth restriction
NT	Nuchal translucency
AFI	Amniotic fluid index
PI	Pulsatility index
NIPT	Non-invasive prenatal testing
BP	Blood pressure
IPAQ	International Physical Activity Questionnaire
EQ-5D-5L	EuroQol 5-Dimension 5-Level Questionnaire
STAI	State-Trait Anxiety Inventory
GME-Q	Glucose Monitoring Experiences Questionnaire
PSQI	Pittsburgh Sleep Quality Index
CSV	Comma-Separated Values
SPE	Studiecentrum voor Perinatale Epidemiologie
IQR	Interquartile range
MAR	Missing-at-random
UZ Leuven	University Hospitals Gasthuisberg Leuven
ICH-GCP	International Conference on Harmonisation–Good Clinical Practice
REDCap	Research Electronic Data Capture
EC	Ethical committee
