# Supplementary material for: Continuous Glucose Monitoring for Women with Gestational Diabetes, CORDELIA: A Multicentric Randomized Controlled Trial, Methodology and Design
Source: J Clin Med. 2026 Sep 29;15(19):7581. doi: 10.3390/jcm15197581 (PMC13665732; doi:10.3390/jcm15197581)
Supplement: Supplementary file 1 [file jcm-15-07581-s001.zip › jcm-4460511-supplementary.pdf]

Consent form: data collection by dropout after randomization

Title of the study: Continuous Glucose Monitoring for women with gestational diabetes: a randomized controlled trial

Name of the study: The CORDELIA study

Sponsor: UZ Leuven, Herestraat 49, 3000 Leuven

Name and contact details of the investigators:

UZ Leuven, Herestraat 49, 3000 Leuven: Endocrinology: Prof Katrien Benhalima and Prof. Chantal Mathieu

UZA, Drie Eikenstraat 655, 2650 Edegem: Endocrinology: Dr Niels Bochanen and Dr Jolijn Van Cauwenberghe

ZNA, Lindendreef 1, 2020 Antwerpen: Endocrinology: Dr Astrid Morrens and Dr Greet Peeters

OLV Aalst-Asse, Moorselbaan 164, 9300 Aalst: Endocrinology: Dr Inge Van Pottelbergh and Dr Liesbeth Van Nuffel

Imelda Bonheiden, Imeldalaan 9, 2820 Bonheiden: Endocrinology: Dr Dahae Lee and Dr Toon Maes

UZ Gent, Corneel Heymanslaan 10, 9000 Gent: Endocrinology: Dr Joke Marlier and Prof Bruno Lapauw

AZ Groeninge, President Kennedylaan 4, 8510 Kortrijk: Endocrinology: Dr Ellen Heyns and Dr Gert-Jan Vereecke

AZ St-Jan Brugge, Ruddershove 10, 8000 Brugge: Endocrinology: Dr Sara Vandewalle and Dr Anaïs De Landsheer

Vitaz, Moerlandstraat 1, 9100 Sint-Niklaas: Endocrinology: Dr Peter Coremans and Dr Dashty Husein

AZ St Maarten Mechelen, Liersesteenweg 435, 2800 Mechelen: Endocrinology: Dr Kathleen Bollaerts and Dr Anne-Marie Vandenbroucke

AZ St Lucas Gent, Groenebriel 1, 9000 Gent: Endocrinology: Dr Inge Van Boxelaer and Dr Birgit Janssens

Erasme, Lenniksebaan 808, 1070 Bruxelles: Endocrinology: Dr Tiphaine Carton and prof Laurent Crenier

CHU de Liège, Avenue de l'Hôpital 1, 4000 Liège : Endocrinology: Prof Jean-Christophe Philips and Dr Sébastien Grandfils

AZ Oostende, campus Damiaan, Konterdamkaai, 8400 Oostende: Endocrinology: Dr Linsey Winne and Dr Greet Roef

Royal Prince Alfred Hospital/Sydney LHD, Missenden Road CAMPERDOWN NSW 2050, Australia: Endocrinology: Dr Arianne Sweeting and Dr Glynis Ross

Campbelltown Hospital/SWSLHD, 1 Therry Road CAMPBELLTOWN NSW 2560, Australia; Endocrinology: Prof David Simmons and Dr Ivan Kuo

Name and contact details of the local investigators: XX

Name	Function	In case of	Contact details
	Principal investigator of the site	Information, problems or concerns	
	The trial staff	Information, problems or concerns	
	Emergency contact [not emergency department of hospital]	Emergency	
	Patient rights ombudsman	Concerns relating to your rights as a participant in a trial	
Name and address of insurance company of the sponsor & contact of insurer		In case of disagreement or complaint on a damage claim	
	Data protection officer of the site	Questions relating to the condifentiality of your data	
	Belgian Data Protection Authority	Complaints relating to the confidentiality of your data	

Why do we ask your permission to collect data after your participation in the CORDELIA study was stopped prematurely?

You or your physician have decided to prematurely stop your participation in the study. In the context of this study, it is crucial that the data of all participants, regardless of their duration of participation, are included in the analysis of the results. This is because we apply the intention-to-treat principle. This principle means that we analyse results based on the participants' original treatment assignment, rather than only on the treatment actually received. This ensures a fair and accurate evaluation of treatment effectiveness and safety, as the possible effect of stopping prematurely is included in the analysis. To collect your data up to and including the fasting sugar test, which takes place according to the usual routine 6-24 weeks after delivery, we need your consent.

How do we collect your data?

If you consent by signing this form, your data will be collected through your electronic medical record. This will include data from routine care, HbA1c values, gestational diabetes treatment, sensor and/or finger prick data, pregnancy outcomes and the results of your comprehensive fasting sugar test 6-24 weeks after the delivery. Under no circumstances will further study-specific actions be performed or will you be questioned as part of the study.

Free participation

Giving consent is entirely voluntary; there can be no pressure in any way. Do not hesitate to ask any questions that come to mind before signing. Take the time to discuss it with a counselor if you wish. You have the right not to participate or to discontinue this study without having to give any reason even if you have previously agreed to participate in this study. Under no circumstances will your decision affect your relationship with the investigator, nor the quality of your further care. If you agree to participate in this study, sign the consent form. The investigator will also sign this form and will confirm that he has provided you with the necessary information about this study. You will receive the copy intended for you.

The confidentiality is guaranteed

Your data will be processed in accordance with the European General Data Protection Regulation (AVG / GDPR) and Belgian Law (Belgian Law 30 Jul 2018) on the protection of natural persons with regard to the processing of personal data. As the responsible entity of the research, UZ Leuven is the controller of your personal coded data that is processed in the context of the research. The data collected for the study will be archived by the centers according to local practice, and for a period of at least 10 years after the study has ended.
Your participation in the study means that the physician investigator collects your data and uses it for medical publications. You also have the right to access your personal information through the researcher and to have any necessary improvements made to it[1]. The researcher has a duty to treat the collected data confidentially. This means that he ensures never to publish your name in the context of a publication or in the context of a conference. The study

data are always coded before it is transferred to third parties. This code (link between the participant and her data) will be kept by the research team. The researcher is therefore the only person who can establish a link between code and participant[2]. The transferred personal data does not include a combination of elements that allow to identify you[3].

Under strict conditions, under the responsibility of the investigator and under his/her supervision (or that of one of his/her research assistants), direct access to the medical record may be granted to authorized personnel of the sponsor or of its representative(s), to regulatory authorities or to other persons authorized by law to verify that the study is being performed correctly (such as representatives of ethics committees or an external auditing company). Access is only granted to check the quality of the collected data as part of the study. All persons who are allowed to inspect the medical file are bound by professional secrecy. Therefore, your consent to participate in this study also means that your encrypted medical data is used for purposes described in this information form and that it is transferred to the above persons and / or institutions.

The processing of your personal data is necessary in order to be able to realize the scientific research purposes as described. Conducting academic research is one of UZ Leuven's statutory assignments. As a university hospital affiliated with KU Leuven, UZ Leuven must support science and education in the public interest. UZ Leuven wants to clarify the necessity of processing as part of scientific research and as a task of general interest, as the legal basis for processing data by UZ Leuven in the context of this research. In addition, UZ Leuven is subject to specific legal obligations that govern the processing of your data which may be necessary in the context of safety reporting. For data protection legislation, we follow the European GDPR (General Data Protection Regulation) regulations. According to the GDPR, you have the right to inspect the processing of your data. If you have any questions about this, please contact the data protection officer of the study center. The contact details of the local Data Protection Agency are (…).

You have the right to file a complaint about how your information is handled, with the Belgian supervisory authority responsible for enforcing the data protection legislation:
Data Protection Authority
Rue de la Presse 35
1000 Brussels
Tel. +32 2 274 48 00
e-mail: contact(at)apd-gba.be
Website: www.dataprotectionauthority.be

[1]These rights are determined by the European General Data Protection Regulation (GDPR) and by the law of August 22, 2002 on patient rights.

[2] For clinical studies, the law requires that you maintain a record of your record for 20 years. In the case of a study medicine for an innovative therapy that uses human body material, this period is a minimum of 30 years and a maximum of 50 years in accordance with the Belgian law of 19 December 2008 on the use of human body material and the applicable Royal Decrees.
[3]The examination results database therefore does not relate to elements such as your initials, your gender and your full date of birth (dd / mm / yyyy).

Informed Consent

Consent form: data collection after dropout after randomization

The responsible person has clearly given me all the necessary information on the aims of the data collection. I have received sufficient time to read the text, to think about it and to talk about it with somebody of my choice. I clearly understand that my participation is entirely voluntary and that at any time I can prematurely end my participation without that this will influence the way I will be treated in the future.

□ I accept/do not accept (delete as appropriate) that my data will be collected through my electronic medical record during the remainder of the pregnancy up to and including the sugar test 6-24 weeks after the delivery.

……………………………………………	……………………………………………	……………………
First name + name patient	Signature patiënt	Date
……………………………………………	……………………………………………	……………………
First name + name responsible person	Signature responsible person	Date

Made in 2 copies: 1 for the patient and 1 for the doctor

Informed Consent

Consent form: data collection after dropout after randomization

The responsible person has clearly given me all the necessary information on the aims of the data collection. I have received sufficient time to read the text, to think about it and to talk about it with somebody of my choice. I clearly understand that my participation is entirely voluntary and that at any time I can prematurely end my participation without that this will influence the way I will be treated in the future.

□ I accept/do not accept (delete as appropriate) that my data will be collected through my electronic medical record during the remainder of the pregnancy up to and including the sugar test 6-24 weeks after the delivery.

…………………………………………	…………………………………………	……………………
First name + name patient	Signature patiënt	Date
…………………………………………	…………………………………………	……………………
First name + name responsible person	Signature responsible person	Date

Made in 2 copies: 1 for the patient and 1 for the doctor

Title of the study: Continuous Glucose Monitoring for women with gestational diabetes: a randomized controlled trial

Name of the study: The CORDELIA study

Sponsor: UZ Leuven, Herestraat 49, 3000 Leuven

Trial number: S68870

Registration number ClincalTrials.gov: NCT06310356

Sponsor(s) of the trial: University Hospitals Leuven (UZ Leuven)

Site name: UZ Leuven

Main address of site: UZ Leuven, Herestraat 49, 3000 Leuven

Name and contact details of the investigators:

UZ Leuven, Herestraat 49, 3000 Leuven: Endocrinology: Prof Katrien Benhalima and Prof. Chantal Mathieu

UZA, Drie Eikenstraat 655, 2650 Edegem: Endocrinology: Dr Niels Bochanen and Dr Jolijn Van Cauwenberghe

ZNA, Lindendreef 1, 2020 Antwerpen: Endocrinology: Dr Astrid Morrens and Dr Greet Peeters

OLV Aalst-Asse, Moorselbaan 164, 9300 Aalst: Endocrinology: Dr Inge Van Pottelbergh and Dr Liesbeth Van Nuffel

Imelda Bonheiden, Imeldalaan 9, 2820 Bonheiden: Endocrinology: Dr Dahae Lee and Dr Toon Maes

UZ Gent, Corneel Heymanslaan 10, 9000 Gent: Endocrinology: Dr Joke Marlier and Prof Bruno Lapauw

AZ Groeninge, President Kennedylaan 4, 8510 Kortrijk: Endocrinology: Dr Ellen Heyns and Dr Gert-Jan Vereecke

AZ St-Jan Brugge, Ruddershove 10, 8000 Brugge: Endocrinology: Dr Sara Vandewalle and Dr Anaïs De Landsheer

Vitaz, Moerlandstraat 1, 9100 Sint-Niklaas: Endocrinology: Dr Peter Coremans and Dr Dashty Husein

AZ St Maarten Mechelen, Liersesteenweg 435, 2800 Mechelen: Endocrinology: Dr Kathleen Bollaerts and Dr Anne-Marie Vandenbroucke

AZ St Lucas Gent, Groenebriel 1, 9000 Gent: Endocrinology: Dr Inge Van Boxelaer and Dr Birgit Janssens

Erasme, Lenniksebaan 808, 1070 Bruxelles: Endocrinology: Dr Tiphaine Carton and prof Laurent Crenier

CHU de Liège, Avenue de l'Hôpital 1, 4000 Liège : Endocrinology: Prof Jean-Christophe Philips and Dr Sébastien Grandfils

AZ Oostende, campus Damiaan, Konterdamkaai, 8400 Oostende: Endocrinology: Dr Linsey Winne and Dr Greet Roef

Royal Prince Alfred Hospital/Sydney LHD, Missenden Road CAMPERDOWN NSW 2050, Australia: Endocrinology: Dr Arianne Sweeting and Dr Glynis Ross

Campbelltown Hospital/SWSLHD, 1 Therry Road CAMPBELLTOWN NSW 2560, Australia; Endocrinology: Prof David Simmons and Dr Ivan Kuo

Name and contact details of the local investigators:

Name	Function	In case of	Contact details
	Principal investigator of the site	Information, problems or concerns	
	The trial staff	Information, problems or concerns	
	Emergency contact [not emergency department of hospital]	Emergency	
	Patient rights ombudsman	Concerns relating to your rights as a participant in a trial	
Name and address of insurance company of the sponsor & contact of insurer		In case of disagreement or complaint on a damage claim	
-	Data protection officer of the **site**	Questions relating to the condifentiality of your data	
-	Belgian Data Protection Authority	Complaints relating to the confidentiality of your data	

I Necessary information for your decision to participate

Introduction

You are invited to participate in a large multicentric study on the use of glucose sensors to measure sugar levels in a continuous manner in pregnant women with gestational diabetes. Gestational diabetes is a type of diabetes that develops during pregnancy. It is important to timely detect and treat gestational diabetes because women with gestational diabetes have a higher risk of complications in pregnancy (such as a higher risk of overweight babies, preeclampsia, preterm birth and caesarean sections). Moreover, women with gestational diabetes also have a greatly increased risk of developing a permanent form of diabetes after delivery (type 2 diabetes). The treatment of gestational diabetes involves following a healthy diet, increasing physical activity (if medically permitted), and monitoring sugar levels through finger pricks (using a glucometer). If adopting a healthy lifestyle alone is insufficient to lower sugar levels, the physician will initiate additional treatment with insulin. With this study, we aim to investigate whether the use of glucose sensors can safely improve control of sugar levels during pregnancy and reduce pregnancy complications. A glucose sensor measures sugar levels under the skin in a continuous manner.

You will not receive any study medication with this study. If you participate in this study, a computer system will decide whether you will be assigned to the group receiving the glucose sensors or to the control group where the standard treatment will continue (where sugar levels are measured with finger pricks through a glucometer). As a participant, you have a 50% chance of being assigned to the group that receives the glucose sensors (intervention group) and you have an equal chance (50%) of being assigned to the control group where the standard treatment is continued.

Before you decide to participate, we ask you to take the time to ask any questions you would like to the physician researcher or study assistant. We therefore ask you to take a thorough look at the practical implications, benefits and possible risks that may be involved, so that you can make an informed decision This study takes place at UZ Leuven, UZA, UZ Gent, Imelda Bonheiden, OLV Aalst-Asse, AZ St Jan Brugge, AZ Groeninge Kortrijk, Vitaz St Niklaas, AZ St Lucas Gent, AZ St Maarten Mechelen, ZNA, Erasme, CHU Liége and AZ Oostende. Recruitment in Australia will be performed in the Campbelltown Hospital/SWSLHD (Lead) and Royal Prince Alfred Hospital.

For this academic study, financial support was obtained from a medical equipment company (Abbott). In addition, medical equipment to measure sugar levels is provided by medical equipment companies (glucose sensors by Abbott and glucometers by Lifescan for Belgium).

If you participate in this clinical trial, you should know the following:

- This clinical study is being initiated after evaluation by several ethics committees.
- Your participation is voluntary; there can be no compulsion in any way. Your signed permission is required to participate. Even after you have signed, you can let the physician researcher know that you want to stop your participation. The decision

whether or not to participate will not have any negative impact on the quality of the care or on the relationship with the treating physician(s).

- The data collected in the context of your participation is confidential. This means that the physician researcher will never disclose your name in the context of a publication or in the context of a conference.
- Insurance is available in the event that you should be harmed as a result of your participation in this clinical study.
- The glucose sensors and measuring equipment with glucometer specifically used for the study are made available free of charge by the sponsor. You will not receive any additional compensation for participating in this study.
- If you require additional information, you can always contact the physician researcher or a member of his or her team.

Additional information about "Rights of the clinical study participant" can be found in attachment on page 15.

The objective of the study

With this study, we want to investigate whether the use of glucose sensors can improve control of sugar levels during pregnancy and reduce pregnancy complications. Additionally, with this study we also want to investigate whether the use of glucose sensors can also be useful to better predict the risk of diabetes 6-24 weeks after the delivery. In this study, half of all participants will receive glucose sensors, while the other half will continue with the current treatment for monitoring sugar levels in gestational diabetes (control group). The current method of monitoring sugar levels in gestational diabetes involves using a glucometer, which measures sugar levels through a finger prick. A glucometer is a device that measures sugar levels using a blood drop obtained through a finger prick.

The estimated number of participants in the study is 386. Before you are assigned to either the group with glucose sensors or the control group, you will be asked to wear a blinded glucose sensor for 1 week so that sugar levels can be measured in a standardized manner before starting with glucose sensors. During this period, in line with the normal routine, you will monitor your sugar levels with finger pricks (using a glucometer). To evaluate sugar levels in a standardized manner between the group with glucose sensors and the control group, participants in the control group will be asked to wear a blinded glucose sensor for a maximum of three times during pregnancy for 2 weeks. A glucose sensor continuously measures sugar levels under the skin. A blinded glucose sensor means that you cannot see the measured sugar levels. While using the blinded sensor, monitoring will continue as usual, and you will be asked to regularly check sugar levels with a glucometer (using finger pricks).
The study starts after gestational diabetes was diagnosed in pregnancy, this can be up to 30 weeks of pregnancy. As a participant, you have a 50% chance of being assigned to the group that receives the glucose sensors (intervention group) and you have an equal chance (50%) of being assigned to the control group where the standard treatment is continued.

The study ends 6-24 weeks after delivery where, in line with normal routine, a comprehensive fasting sugar test is performed to check whether you have persistently elevated sugar levels after the delivery. All participants (regardless of whether you were assigned to the group with glucose sensors or the control group during pregnancy) will also be asked to wear a blinded glucose sensor for 2 weeks during the sugar test specifically for the study. This aims to determine if a glucose sensor can be used as a replacement for the comprehensive fasting sugar test to identify those at highest risk of elevated sugar levels after the delivery.

Who can participate in the study?

To participate, you must have gestational diabetes, be at least 18 years old, and be pregnant with a singleton pregnancy with gestational age up to 29 weeks and 6 days. You must also speak Dutch, French or English and have access to e-mail so that we can easily reach you.

You cannot participate if you have been diagnosed with pre-existing diabetes or have antibodies in your blood that show you are at increased risk of developing type 1 diabetes, if you have a physical or psychological condition that would prevent the study from being conducted (according to your treating physician's advice) or if you are taking medication that can interfere with glucose metabolism (such as use of high doses of cortisone or use of medication for diabetes). You can also not participate if you have undergone weight loss surgery in the past (because gestational diabetes cannot be diagnosed through normal tests) or if you have a multiple pregnancy because multiple pregnancies (even in women without gestational diabetes) have a higher risk of pregnancy complications. You can also not participate if you have a known allergy to the adhesive material used to attach the glucose sensor.

Overview of the study

If you agree to participate, a computer system will decide whether you will be assigned to the group receiving the glucose sensors or to the control group where your current monitoring of sugar levels with finger pricks (using a glucometer) will continue. Before you can be assigned to a group, you will need to wear a blinded glucose sensor for 7 days. Monitoring during the study will follow the normal routine with a check-up at the diabetes consultation every 4-6 weeks. If the physician determines that sugar levels remain too high despite adjusting your diet and increasing physical activity, the physician will initiate treatment with insulin (this is done as needed regardless of whether you are assigned to the glucose sensor group or the control group). Insulin is administered through one or more subcutaneous injections. You will then be taught to administer these injection(s) to yourself. If insulin is started, follow-up at the diabetes consultation will become more frequent (every 2 weeks on average). In most women with gestational diabetes, insulin can be stopped immediately after the delivery.

In line with normal routine, a comprehensive fasting sugar test (oral glucose tolerance test) will be scheduled 6-24 weeks after the delivery, to assess whether you have persistently elevated sugar levels (prediabetes or diabetes). This test is therefore not specific to the study (and would also be performed if you were not participating in the study). For this test, you should come fasting (have not eaten anything for 8-10 hours before the test, you may drink water). First a fasting blood sample will be taken. Then you will be asked to drink a sugar drink in 5 min (you may drink water with this) and blood will be taken again after 2 hours to determine the blood sugar value. If the comprehensive fasting test cannot take place (for example due to nausea and vomiting), a fasting blood sample can alternatively be taken with determination of the blood sugar value and the HbA1c. HbA1c is an analysis in the blood that shows the average sugar regulation over the past 3 months.

<u>If you are assigned to the group with glucose sensors (intervention group)</u>:

- You will receive instructions on how to use the glucose sensor (the FreeStyle Libre 3/or 3 plus by Abbott). You will be taught how to place the glucose sensor on your upper arm. This glucose sensor has already been approved for use during pregnancy. The glucose sensor continuously measures sugar levels under the skin for a maximum of 2 weeks. Every 2 weeks, you will need to replace the glucose sensor. You will be able to read the sugar levels on your smartphone (Android or iOS) using an app (FreeStyle Libre 3-BE), or if you do not have a compatible smartphone, you will be given a reader. When using the glucose sensor, you normally do not need to do finger pricks to measure sugar values. Only if there is a technical problem with the glucose sensor, you will be advised to check the sugar values by finger prick (using a glucometer). If the glucose sensor does no longer measure correctly, you should replace it with a new glucose sensor. If you continue to have problems with the glucose sensors or no longer have sufficient glucose sensors, you should contact a diabetes educator at your center so that new glucose sensors can be provided to you.
- At a maximum of three moments in pregnancy [before allocation, between 31-34 weeks and between 36-39 weeks] and between 6-24 weeks after delivery, you will be asked to complete several questionnaires
- As sugar levels normalize quickly after the delivery, the use of the glucose sensor will be stopped when you leave the hospital after the delivery.

<u>If you are assigned to the control group (with continuation of standard care)</u>:

- Your current monitoring of sugar levels by glucometer and finger pricks will continue. If your sugar levels are under control by following a healthy diet, you will be asked to measure your sugar levels daily (fasting and 1 or 2 hours after each meal). If additional insulin is started, you will also be asked to measure your sugar levels daily (at least fasting and 1 or 2 hours after each meal). As sugar levels normalize quickly

after the delivery, measuring your sugar levels will be stopped when you leave the hospital after the delivery.

- At a maximum of three moments in pregnancy [between 20-24 weeks (only if gestational diabetes was diagnosed before 20 weeks of pregnancy), between 31-34 weeks and between 36-39 weeks], you will be asked to wear a blinded glucose sensor (Abbott's FreeStyle Libre Pro IQ sensor) for 2 weeks. The glucose sensor will be placed by the diabetes educator on your upper arm during your routine diabetes consultation visit. While using the blinded sensor, your monitoring of sugar levels through a glucometer continues as usual. At different points in pregnancy (at the time of allocation, between 31-34 weeks and between 36-39 weeks of pregnancy) and between 6-24 weeks after delivery, you will be asked to complete several questionnaires specifically for the study.

<u>The further examinations (whether you are assigned to the glucose sensor group or the control group) are the following:</u>

- No additional blood will be collected specifically for the study. In line with normal follow-up (which would also occur if you were not participating in this study), a blood sample will be taken every 4-6 weeks during pregnancy at each participating center to determine the HbA1c level. The HbA1c level and your cholesterol levels will also be determined during the comprehensive fasting sugar test, which takes place in line with normal routine 6-24 weeks after the delivery. As part of the study, you will be asked at a maximum of three moments in pregnancy and once after the delivery to complete max. 12 questionnaires (8 questionnaires at allocation, 3 questionnaires between 31-34 weeks, 12 questionnaires between 36-39 weeks, and 9 questionnaires between 6-24 weeks after the delivery). These questionnaires will be on diet, physical activity, depression, anxiety, general quality of life and sleep quality, fear of low or high sugar levels, satisfaction regarding treatment of gestational diabetes, satisfaction regarding glucose sensor use, and on breastfeeding and contraception after delivery. If the questionnaires show that you have significant symptoms of depression or severe anxiety, you will be offered counseling (as would be done if you did not participate in the study) by a psychologist or psychiatrist in your center.
- At the start of the study, you will also be asked to complete a questionnaire about your socio-economic background, including questions about your origin, ethnicity, education, occupation and income. The collection of these data is important because socio-economic factors are associated with an increased risk of developing gestational diabetes and other pregnancy complications. These socio-economic factors are also associated with a higher risk of developing a persistent form of diabetes after childbirth.
- Completing the questionnaires takes a maximum of 20-30 minutes and can be done during the consultation. If it is more convenient to complete the questionnaire at home, the research team sends a personalised link via REDCap, which is directly linked

to the participant's profile.The clinical examination conducted during the diabetes consultation follows the normal routine, including measurements of your height, weight, and blood pressure. There is no additional clinical examination as part of the study.

- The clinical examination of the baby at birth includes the following routine examinations (not specific to the study): measurement of birth weight, height and head circumference.
- Data will be collected from your medical records regarding your medical history, treatment, pregnancy follow-up, delivery, and follow-up 6-24 weeks after the delivery.
- The research data obtained from this study may be used later along with research data from other similar studies to better evaluate whether treatment with glucose sensors in gestational diabetes leads to fewer pregnancy complications.
- It is possible that after the end of this study, a follow-up study will be planned to investigate the long-term impact of gestational diabetes on you and your child. This consent form therefore also asks for your permission to be contacted during the 10 years following your participation in this study to obtain additional information about your health and that of your child.

The glucose sensor data used for the regular monitoring of gestational diabetes will be uploaded to the digital platform of the sensor’s company (Abbott), either directly through the sensor or via your center's study team. The sensor’s company has no access to any personal data, only to the measured sugar values. The central study team at UZ Leuven will then extract these data from the digital platform for use in the study to make calculations of the measured glucose values, such as how often the measured sugar values are within the target range, or are just below the target range or above the recommended targets.
Additionally, UZ Leuven study team will also centrally process the data from the blinded glucose sensor. Your centre's study team will therefore provide the blinded glucose sensor to the UZ Leuven study team. Some calculations of the measured sugar values can be centrally processed by the sensor company (Abbott) and then provided to the coordinating study team. For the possible processing of the glucose sensor data, an agreement was concluded between the client and the glucose sensor company (Abbott). The data from finger prick measurements, are digitally uploaded via the glucometer platform (either by yourself or by your centre's study team). In addition, coded participant data may be shared with other universities, hospitals and/or commercial partners for analysis in the context of this study.

The summary in the table provides an overview of the examinations that are part of the normal follow-up and the examinations that are specific to the study.

	First visit (after diagnosis)	20-24W	31-34W	36-39W	Delivery	6-24 weeks after the delivery
**Part of normal routine**						
Diabetes consultation (every 2-6 weeks)	X	X	X	X	X	
Use of glucometer for 1 week	X					
Glucometer use (for control group)		X	X	X		
Blood test with HbA1c	X	X	X	X		
Clinical examination mother	X	X	X	X		X
Reading and analysing data of glucose sensor or glucometer and treatment		X	X	X	X	
Clinical examination baby					X	
Fasting sugar test and determination of HbA1c and cholesterol						X
**Specifically related to the study**						
Blinded glucose sensor for 7 days	X					
Blinded glucose sensor for 2 weeks (for control group)		X	X	X		
Blinded glucose sensor for 2 weeks for all participants						X
Continuous use of glucose sensor (for intervention group)	X	X	X	X		
Data collection from the electronic medical record		X	X	X	X	X

Completing questionnaires	X		X	X		X

W: weeks; Yellow: Wearing blinded glucose sensor between 20-24 weeks is only necessary if gestational diabetes was diagnosed <20 weeks

Risks and inconveniences related to the study

No study medication is used in this study. When using glucose sensors, technical problems may occur that cause the glucose sensor to malfunction. If this occurs, you will be able to monitor your sugar levels via finger pricks until a new glucose sensor can be placed. The (blinded) glucose sensor can sometimes cause local skin irritation with redness and itching at the insertion site. If this is due to an allergy to certain materials such as stickers, alternative stickers will be suggested. If glucose sensors are used (intervention group), alarms can be set that alert you when your sugar levels are too high or too low. This may sometimes involve alarms occurring too frequently. If necessary, the alarms will be adjusted so that they do not disturb you unnecessarily.

Advantages

It is possible that there are no benefits for you when participating in this study. On the other hand, it is also possible that you can obtain a limited benefit by participating in the study, by achieving better control of the sugar levels and possibly also fewer pregnancy complications. The information obtained through this study will help us to develop better guidelines for the monitoring and treatment of gestational diabetes.

The communication of new available information

It is possible that during the study, new important information becomes available about the topic we are investigating. You will be informed of any new information that might affect your decision about your participation in the study. In that case, you will be asked either to sign a supplement to the consent form or to sign a new information and consent document. When you decide, after reviewing the new information, to end your participation in the study, the investigator will assure that you will continue to receive the best possible care.

Alternative care

If you decide not to participate and regardless of the reason for your refusal, the attending physician will always ensure that you receive the best possible follow-up.

Stopping your participation in the study early

Your participation in the study is completely voluntary and you have therefore the right to cancel your participation in the study at any time for any reason. It may also sometimes be that the doctor decides to end your participation in the study early for medical reasons or having to start taking medication that affects your sugar levels. The data collected so far will be used for analysis. If you terminate your participation early in the study after

randomization, data in the context of the study during the further course of the pregnancy up to and including hospitalization after delivery can be collected via the electronic patient file if you give permission for this by signing a modified consent form. This includes data from routine care, HbA1c values, treatment, sugar values and pregnancy outcomes. Under no circumstances will any study-specific actions be carried out.

Treatment after stopping the study

When stopping the study early or ending the study normally, the investigator will ensure that the best available treatment is continued with the doctor of your choice.

You should also know that: For your safety, it is important that your general practitioner and / or other health professionals are informed about your participation in this study.

Contact

When you require additional information but also in case of any problems or concerns, you can contact the investigator_____or diabetes-nurse _____. You must also report that you are participating in this study. Your medical record will contain information about the study that may be useful for the doctor on call. When you have questions concerning your rights as a patient, you can contact the mediation service of your hospital at the following telephone number ____.

II Informed consent

Informed consent of 'Continuous measurement of sugar levels in women with gestational diabetes: a randomized controlled trial: the CORDELIA study'

Doctor........................ has clearly given me all the necessary information on the aims, the duration, the different steps of the study and what is expected from me. The advantages and disadvantages and possible inconveniences of a participation were clearly explained. I have received sufficient time to read the text, to think about it and to talk about it with somebody of my choice. I clearly understand that my participation is entirely voluntary and that at any time I can prematurely end my participation without that this will influence the way I will be treated in the future. I understand that during my participation in this study, data will be collected about me and that the doctor and the sponsor ensure the confidentiality of these data in accordance with the relevant European and Belgian legislation.
I understand that the execution of this study by UZ Leuven serves the public interest and the processing of my personal data is necessary for the execution of this study.

□ I accept/do not accept (delete as appropriate) that the investigator informs me and my treating physician of results and the possible consequences of an accidental finding during the study.

□ I accept/do not accept (delete as appropriate) that I can be contacted up to 10 years after the end of my participation in the study to determine whether you wish to participate in a follow-up study to obtain additional data on my and my baby's health.

....	....	....
First name + name patient	Signature patiënt	Date
....	....	....
First name + name responsible doctor	Signature doctor	Date

Made in 2 copies: 1 for the patient and 1 for the doctor

Informed consent of 'Continuous measurement of sugar levels in women with gestational diabetes: a randomized controlled trial: the CORDELIA study'

Doctor........................ has clearly given me all the necessary information on the aims, the duration, the different steps of the study and what is expected from me. The advantages and disadvantages and possible inconveniences of a participation were clearly explained. I have received sufficient time to read the text, to think about it and to talk about it with somebody of my choice. I clearly understand that my participation is entirely voluntary and that at any time I can prematurely end my participation without that this will influence the way I will be treated in the future. I understand that during my participation in this study, data will be collected about me and that the doctor and the sponsor ensure the confidentiality of these data in accordance with the relevant European and Belgian legislation.
I understand that the execution of this study by UZ Leuven serves the public interest and the processing of my personal data is necessary for the execution of this study.

□ I accept/do not accept (delete as appropriate) that the investigator informs me and my treating physician of results and the possible consequences of an accidental finding during the study.

□ I accept/do not accept (delete as appropriate) that I can be contacted up to 10 years after the end of my participation in the study to determine whether you wish to participate in a follow-up study to obtain additional data on my and my baby's health.

....	....	....
First name + name patient	Signature patiënt	Date
....	....	....
First name + name responsible doctor	Signature doctor	Date

Made in 2 copies: 1 for the patient and 1 for the doctor

III Additional information

1. Accidental findings

A result that is found during the study and on top of the objectives is called an accidental finding. If this result could be important for your health or that of your blood relatives, the sponsor will inform the researcher about this. With your permission, the researcher will inform you and your treating physician of your results and the possible consequences. If necessary, the investigator and / or the treating physician will advise you on what to do.

2. Additional information about the protection and rights of clinical trial participants

The Ethics Committee

This study was approved by an independent ethics committee, in particular the Ethics Committee UZ / KU Leuven, which operates as the central ethics committee, after obtaining approval of the local ethics committees of each participating center in Belgium. This study is conducted in line with the directives of good clinical practice (ICH/GCP) and according to the most recent version of the declaration of Helsinki, drawn up to protect the rights of subjects participating in clinical studies. Under no circumstances should you consider the approval by the Medical Ethics Committee as an incentive to participate in this study.

Free participation

Do not hesitate to ask any questions that come to mind before signing. Take the time to discuss it with a counselor if you wish.

You have the right not to participate in or to discontinue this study without having to give any reason even if you have previously agreed to participate in this study. Under no circumstances will your decision affect your relationship with the investigator, nor the quality of your further care.

If you agree to participate in this study, sign the consent form. The investigator will also sign this form and will confirm that he has provided you with the necessary information about this study. You will receive the copy intended for you.

For your safety, it is recommended to notify the investigator if you decide to discontinue your study.

Costs associated with your participation

The sponsor will reimburse costs specific to the study . This means that if you decide to participate in this study, the glucose sensors and glucose monitoring equipment used specifically for this study will be provided to you without charge by the sponsor. Therefore, there is no additional cost to you or your health insurance company to participate in this study. You will not receive any additional compensation for your participation in this study.The confidentiality is guaranteed

Your data will be processed in accordance with the European General Data Protection Regulation (AVG / GDPR) and Belgian Law (Belgian Law 30 Jul 2018) on the protection of natural persons with regard to the processing of personal data. As the responsible entity of the research, UZ Leuven is the controller of your personal data that is processed in the context of the research.

The data collected for the study will be archived by the centers according to local practice, and for a period of at least 10 years after the study has ended. Collecting data for 10 years is essential to enable follow-up studies.

Your participation in the study means that the physician investigator collects your data and uses it for medical publications. You also have the right to access your personal information through the researcher and to have any necessary improvements made to it[1]. The researcher has a duty to treat the collected data confidentially. This means that he ensures never to publish your name in the context of a publication or in the context of a conference. The study data are always coded before it is transferred to third parties. This code (link between the participant and her data) will be kept by the research team. The researcher is therefore the only person who can establish a link between code and participant[2]. The transferred personal data does not include a combination of elements that allow to identify you[3]. The sponsor then processes this coded data under strict confidentiality rules.

Under strict conditions, under the responsibility of the investigator and under his/her supervision (or that of one of his/her research assistants), direct access to the medical record may be granted to authorized personnel of the sponsor or of its representative(s), to regulatory authorities or to other persons authorized by law to verify that the study is being performed correctly (such as representatives of ethics committees or an external auditing company). Access is only granted to check the quality of the collected data as part of the study. All persons who are allowed to inspect the medical file are bound by professional secrecy. Therefore, your consent to participate in this study also means that your encrypted medical data is used for purposes described in this information form and that it is transferred to the above persons and / or institutions.

The processing of your personal data is necessary in order to be able to realize the scientific research purposes as described. Conducting academic research is one of UZ Leuven's statutory assignments. As a university hospital affiliated with KU Leuven, UZ Leuven must support science and education in the public interest. UZ Leuven wants to clarify the necessity of processing as part of scientific research and as a task of general interest, as the legal basis for

processing data by UZ Leuven in the context of this research. In addition, UZ Leuven is subject to specific legal obligations that govern the processing of your data which may be necessary in the context of safety reporting (such as, for example, reporting adverse reactions to supervisory authorities).

For data protection legislation, we follow the European GDPR (General Data Protection Regulation) regulations. According to the GDPR, you have the right to inspect the processing of your data. If you have any questions about this, please contact the data protection officer of the study center. The contact details of the local Data Protection Agency are __________.

You have the right to file a complaint about how your information is handled, with the Belgian supervisory authority responsible for enforcing the data protection legislation:

Data Protection Authority
Rue de la Presse 35
1000 Brussels
Tel. +32 2 274 48 00
e-mail: contact(at)apd-gba.be
Website: www.dataprotectionauthority.be

Insurance for the Belgian centers: The sponsor, UZ Leuven, is in line with the Belgian law of 7 May 2004 concerning the experiments on humans, even faultless, liable for all damage that occurs to the participant or his right holders, directly or indirectly related to the study[4]. As participant to this study you are covered by the insurance policy UZ/KU Leuven has completed at the insurance broker Vanbreda Risk & Benefits NV, Plantin en Moretuslei 297, 2140 Antwerp, under the policy number 299.053.700. You can obtain a copy of the insurance policy and the conditions through your investigator. The participant or his entitled parties can also summon the insurer directly.

---

[1]These rights are determined by the European General Data Protection Regulation (GDPR) and by the law of August 22, 2002 on patient rights.
[2] For clinical studies, the law requires that you maintain a record of your record for 20 years. In the case of a study medicine for an innovative therapy that uses human body material, this period is a minimum of 30 years and a maximum of 50 years in accordance with the Belgian law of 19 December 2008 on the use of human body material and the applicable Royal Decrees. [3]The examination results database therefore does not relate to elements such as your initials, your gender and your full date of birth (dd / mm / yyyy).
[4]In accordance with article 29 of the Belgian Act on experiments on the human person (7 May 2004)

SPIRIT 2025 checklist of items to address in a randomized trial protocol*

Section / Topic	No	SPIRIT 2025 checklist item description	Reported on page no.
**Administrative information**			
Title and structured summary	1a	Title stating the trial design, population, and interventions, with identification as a protocol	1,3,13
	1b	Structured summary of trial design and methods, including items from the World Health Organization Trial Registration Data Set	8-19
Protocol version	2	Version date and identifier	13
Roles and responsibilities	3a	Names, affiliations, and roles of protocol contributors	1
	3b	Name and contact information for the trial sponsor	1,25
	3c	Role of trial sponsor and funders in design, conduct, analysis, and reporting of trial; including any authority over these activities	25
	3d	Composition, roles, and responsibilities of the coordinating site, steering committee, endpoint adjudication committee, data management team, and other individuals or groups overseeing the trial, if applicable	20-25
**Open science**			
Trial registration	4	Name of trial registry, identifying number (with URL), and date of registration. If not yet registered, name of intended registry	4
Protocol and statistical analysis plan	5	Where the trial protocol and statistical analysis plan can be accessed	19-22
Data sharing	6	Where and how the individual de-identified participant data (including data dictionary), statistical code, and any other materials will be accessible	23-25
Funding and conflicts of interest	7a	Sources of funding and other support (e.g., supply of drugs)	25
	7b	Financial and other conflicts of interest for principal investigators and steering committee members	25
Dissemination policy	8	Plans to communicate trial results to participants, healthcare professionals, the public, and other relevant groups (e.g., reporting in trial registry, plain language summary, publication)	25
**Introduction**			
Background and rationale	9a	Scientific background and rationale, including summary of relevant studies (published and unpublished) examining benefits and harms for each intervention	6-7
	9b	Explanation for choice of comparator	6-7 and 12
Objectives	10	Specific objectives related to benefits and harms	8
**Methods: Patient and public involvement, trial design**			
Patient and public involvement	11	Details of, or plans for, patient or public involvement in the design, conduct, and reporting of the trial	22
Trial design	12	Description of trial design including type of trial (e.g., parallel group, crossover), allocation ratio, and framework (e.g., superiority, equivalence, non-inferiority, exploratory)	

**Methods: Participants, interventions, and outcomes**			
Trial setting	13	Settings (e.g., community, hospital) and locations (e.g., countries, sites) where the trial will be conducted	9-12
Eligibility criteria	14a	Eligibility criteria for participants	35 - table 2
	14b	If applicable, eligibility criteria for sites and for individuals who will deliver the interventions (e.g., surgeons, physiotherapists)	10
Intervention and comparator	15a	Intervention and comparator with sufficient details to allow replication including how, when, and by whom they will be administered. If relevant, where additional materials describing the intervention and comparator (e.g., intervention manual) can be accessed	9-12
	15b	Criteria for discontinuing or modifying allocated intervention/comparator for a trial participant (e.g., drug dose change in response to harms, participant request, or improving/worsening disease)	19
	15c	Strategies to improve adherence to intervention/comparator protocols, if applicable, and any procedures for monitoring adherence (e.g., drug tablet return, sessions attended)	22
	15d	Concomitant care that is permitted or prohibited during the trial	35 table 1 and 19
Outcomes	16	Primary and secondary outcomes, including the specific measurement variable (e.g., systolic blood pressure), analysis metric (e.g., change from baseline, final value, time to event), method of aggregation (e.g., median, proportion), and time point for each outcome	8-11
Harms	17	How harms are defined and will be assessed (e.g., systematically, non-systematically)	11
Participant timeline	18	Time schedule of enrollment, interventions (including any run-ins and washouts), assessments, and visits for participants. A schematic diagram is highly recommended (see Figure)	Figure 2 and table 2
Sample size	19	How sample size was determined, including all assumptions supporting the sample size calculation	19
Recruitment	20	Strategies for achieving adequate participant enrollment to reach target sample size	12
**Methods: Assignment of interventions**			
Randomization:			
Sequence generation	21a	Who will generate the random allocation sequence and the method used	12
	21b	Type of randomization (simple or restricted) and details of any factors for stratification. To reduce predictability of a random sequence, other details of any planned restriction (e.g., blocking) should be provided in a separate document that is unavailable to those who enroll participants or assign interventions	12
Allocation concealment mechanism	22	Mechanism used to implement the random allocation sequence (e.g., central computer/telephone; sequentially numbered, opaque, sealed containers), describing any steps to conceal the sequence until interventions are assigned	12
Implementation	23	Whether the personnel who will enroll and those who will assign participants to the interventions will have access to the random allocation sequence	12

Blinding	24a	Who will be blinded after assignment to interventions (e.g., participants, care providers, outcome assessors, data analysts)	/
	24b	If blinded, how blinding will be achieved and description of the similarity of interventions	/
	24c	If blinded, circumstances under which unblinding is permissible, and procedure for revealing a participant's allocated intervention during the trial	/
**Methods: Data collection, management, and analysis**			
Data collection methods	25a	Plans for assessment and collection of trial data, including any related processes to promote data quality (e.g., duplicate measurements, training of assessors) and a description of trial instruments (e.g., questionnaires, laboratory tests) along with their reliability and validity, if known. Reference to where data collection forms can be accessed, if not in the protocol	22
	25b	Plans to promote participant retention and complete follow-up, including list of any outcome data to be collected for participants who discontinue or deviate from intervention protocols	22
Data management	26	Plans for data entry, coding, security, and storage, including any related processes to promote data quality (e.g., double data entry; range checks for data values). Reference to where details of data management procedures can be accessed, if not in the protocol	22
Statistical methods	27a	Statistical methods used to compare groups for primary and secondary outcomes, including harms	19-22
	27b	Definition of who will be included in each analysis (e.g., all randomized participants), and in which group	19-22
	27c	How missing data will be handled in the analysis	19-22
	27d	Methods for any additional analyses (e.g., subgroup and sensitivity analyses)	19-22
**Methods: Monitoring**			
Data monitoring committee	28a	Composition of data monitoring committee (DMC); summary of its role and reporting structure; statement of whether it is independent from the sponsor and funder; conflicts of interest and reference to where further details about its charter can be found, if not in the protocol. Alternatively, an explanation of why a DMC is not needed	22
	28b	Explanation of any interim analyses and stopping guidelines, including who will have access to these interim results and make the final decision to terminate the trial	19-22
Trial monitoring	29	Frequency and procedures for monitoring trial conduct. If there is no monitoring, give explanation	22
**Ethics**			
Research ethics approval	30	Plans for seeking research ethics committee/institutional review board approval	23
Protocol amendments	31	Plans for communicating important protocol modifications to relevant parties	13
Consent or assent	32a	Who will obtain informed consent or assent from potential trial participants or authorized proxies, and how	23
	32b	Additional consent provisions for collection and use of participant data and biological specimens in ancillary studies, if applicable	19
Confidentiality	33	How personal information about potential and enrolled participants will be collected, shared, and maintained in order to protect confidentiality before, during, and after the trial	23

Ancillary and post-trial care	34	Provisions, if any, for ancillary and post-trial care, and for compensation to those who suffer harm from trial participation	/

*We strongly recommend reading this checklist in conjunction with the SPIRIT 2025 Explanation and Elaboration and the SPIRIT 2025 Expanded Checklist for important clarifications on all the items. We also recommend reading relevant SPIRIT extensions. See www.consort-spirit.org

Citation: Chan A-W, Boutron I, Hopewell S, Moher D, Schulz KF, et al. SPIRIT 2025 statement: updated guideline for protocols of randomised trials. BMJ 2025;389:e081477. https://dx.doi.org/10.1136/bmj-2024-081477

© 2025 Chan A-W et al. This is an Open Access article distributed under the terms of the Creative Commons Attribution License (https://creativecommons.org/licenses/by/4.0/), which permits unrestricted use, distribution, and reproduction in any medium, provided the original work is properly cited.
